# Characterization of four mitochondrial genomes from superfamilies Noctuoidea and Hyblaeoidea with their phylogenetic implications

**DOI:** 10.1038/s41598-022-21502-y

**Published:** 2022-11-07

**Authors:** Rauf Ahmad Shah, Muzafar Riyaz, Savarimuthu Ignacimuthu, Kuppusamy Sivasankaran

**Affiliations:** 1 Division of Taxonomy and Biodiversity, Entomology Research Institute, Loyola Collège, Chennai, Tamil Nadu 600034 India; 2 Xavier Research Foundation, St. Xavier’s College, Palayamkottai, Tamil Nadu 627002 India

**Keywords:** Zoology, Entomology

## Abstract

In the present study, the newly sequenced mitogenomes of three Noctuoid and one Hyblaeoid (Insecta: Lepidoptera) species were annotated based on next-generation sequence data. The complete mitogenome lengths of *Oraesia emarginata, Actinotia polyodon, Odontodes seranensis,* and *Hyblaea puera* were 16,668 bp, 15,347 bp, 15,419 bp, and 15,350 bp, respectively. These mitogenomes were found to encode 37 typical mitochondrial genes (13 protein-coding, 22 transfer RNA, 2 ribosomal RNA) and a control region, similar to most Lepidoptera species. Maximum likelihood (ML) methods and Bayesian inference (BI) were used to reconstruct the phylogenetic relationships of the moths. This study showed the relationships of Noctuoid families as follows: (Notodontidae + (Erebidae + (Nolidae + (Euteliidae + Noctuidae)))). Furthermore, the species *H. puera* was separately clustered from the Noctuoidea member groups. Till now, the species from the superfamily Hyblaeoidea have not been discussed for their phylogenetic relationships. In this study, the complete mitochondrial genome of one species from the superfamily Hyblaeoidea was analysed.

## Introduction

Complete mitochondrial genomes (mitogenomes) have been widely used to infer phylogenetic relationships specifically for wide-ranging groups of organisms such as insects. Mitogenomes have been considerably used as molecular tools for phylogenetic investigations, and comparative and evolutionary relationship studies^1–3^. The insect mitogenomes are relatively small in size, show rapid evolution rates, have low level of recombination, and possess maternal inheritance^4–7^. Therefore, the utilization of mitogenome is expected to provide novel information concerning the classification of insects and assessments of their evolutionary features.

The insect mitochondrial genome is typically a double-stranded, circular molecule that is 14–19 kb in length and is composed of 13 protein-coding genes (PCGs): two ATPase genes (*atp6* and *atp8*), three cytochrome c oxidase genes 1–3 (*cox1-cox3*), one cytochrome B (*cob*), seven NADH dehydrogenase genes (*nad1-6* and *nad4L*), 22 transfer RNA (tRNA), two ribosomal RNA (*rrnL* and *rrnS*) genes and non-coding A + T- rich region^2,5,8–10^.

There are about 1,57,424 described moth species worldwide, from 45 superfamilies, and belonging to 139 families^11^. Analysis of the phylogenetic relationships of lepidopteran moths using mitogenomes has increased rapidly during the last decade^12^. However, the primary focus has been on 11 moth superfamilies namely Bombycoidea, Cossoidea, Gelechioidea, Geometroidea, Hepialoidea, Noctuoidea, Pyraloidea, Tineoidea, Tortricoidea, Yponomeutoidea, and Zygaenoidea.

Noctuoidea is the largest superfamily of the order Lepidoptera, comprising 42, 407 species^11^. The monophyly of Noctuoidea is supported by the existence of a gained apomorphic character, metathoracic tympanal organ^13^. Phylogenetic studies of Noctuoidea were primarily analyzed using molecular methods based on one or two genes and with limited taxon sampling^14–17^. The molecular phylogenetic relationship of Noctuoidea has been analyzed based on single mitochondrial (cox1) and seven nuclear genes (EF-1α, wingless, RpS5, IDH, MDH, GAPDH, and CAD) from 152 species^18^. Zahiri et al.^18^ have proposed a novel perception, separating the traditional group of quadrifid noctuids, and re-establishing Erebidae and Nolidae as families. This result contrasted meaningfully with previous investigations of both morphological and molecular studies. Nevertheless, this analysis failed to resolve the phylogenetic relationships between Erebidae subfamilies^19^. The phylogenetic relationships of the family Erebidae were analyzed using the mitogenomes^20–34^. Several mitogenome sequencing studies were carried out in the family Noctuidae for their phylogenetic utilization[^35–42^] and single mitogenomic analysis was done in the family Euteliidae^12^.

Hyblaeoidea is among the smallest superfamilies in the order Lepidoptera and consists of only one family Hyblaeidae comprising only two genera *Hyblaea* and *Erythrochrus*. The family Hyblaeidae contains only 20 species distributed all over the new and old-world tropics and subtropics. The genus *Hyblaea* is known as a serious forest pest^43^. Twort et al.^44^ have done the whole genome sequence and analyzed the phylogenetic relationships of *Hyblaea puera* and *Hyblaea madagascariensis*. They used the dataset of 162 taxa for analyses, which showed the stable placement of *Hyblaea* as sister to the Pyraloidea member group with strongly supported values.

At present, 84 complete mitogenomes of Noctuoidea from eight families have been deposited in GenBank^12,20,21,24–26,28,30,31,36,37,39,41,45–50^. In the present study, we sequenced the complete mitochondrial genomes of four species representing superfamilies Noctuoidea (*Oraesia emarginata*, *Actinotia polyodon*, *Odontodes seranensis*) and Hyblaeoidea (*Hyblaea puera*) for the first time. The mitogenomes of these species were annotated and the general characteristics of the mitogenome sequences were analyzed and compared. We analyzed phylogenetic relationships of mitogenomes from 90 lepidopteran taxa. In addition, the phylogenetic tree was reconstructed using the maximum likelihood method and Bayesian inference to evaluate the relationships among the lepidopteran moths.

## Results and discussion

### Genome organization and base composition

**Figure 1:**
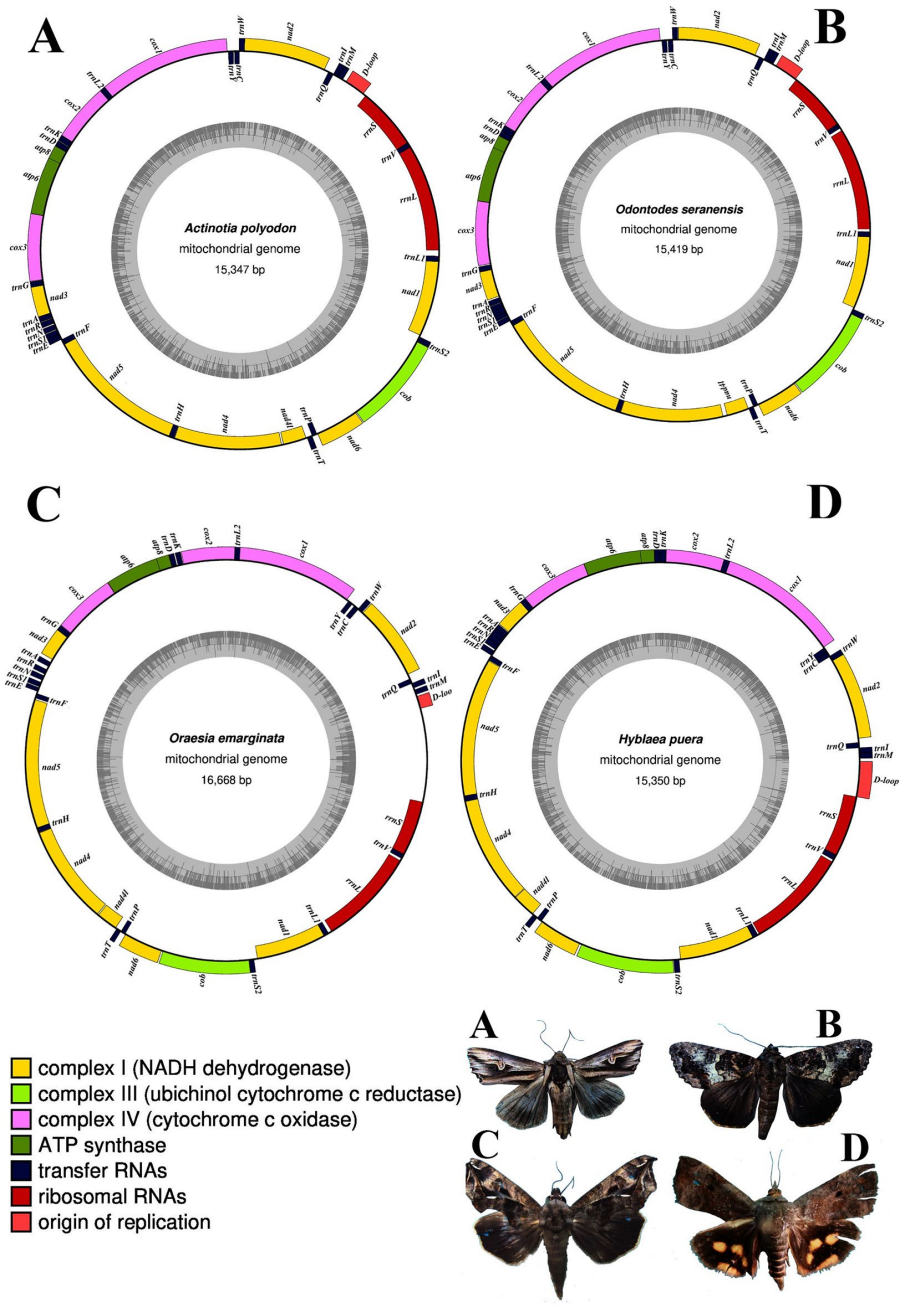
Circular maps of the newly sequenced complete mitochondrial genomes of (**A**) *Actinotia polyodon,* (**B**) *Odontodes seranensis,* (**C**) *Oraesia emarginata,* (**D**) *Hyblaea puera.*

In this study, we sequenced and characterized the complete mitogenomes of three Noctuoidea species; *Oraesia emarginata* (GenBank Accession no. MW648382), *Actinotia polyodon* (GenBank Accession no. MW697903), *Odontodes seranensis* (GenBank Accession no. MW719565) and one Hyblaeoidea species *Hyblaea puera* (GenBank Accession no. MW885970). The sequences were deposited in GenBank (Table 1). The total lengths of the mitogenomes of *O. emarginata*, *A. polyodon*, *O. seranensis,* and *H. puera* were 16, 668 bp, 15, 347 bp, 15, 419 bp, and 15, 350 bp, respectively. The sizes of mitogenome sequenced so far in the superfamily Noctuoidea ranged from 15, 229 bp in *Helicoverpa gelotopoeon* to 16, 346 bp in *Spodoptera frugiperda*. The mitogenome sequence lengths of *A. polyodon*, *O. seranensis,* and *H. puera* fell within the range of mitogenome of other sequenced Noctuoid moths. However, the mitogenome of *Oraesia emarginata* was larger than that of *Gynaephora jiuzhiensis*. The organization of newly sequenced mitogenomes of four species are presented in Fig. 1.

**Table 1:** List of the complete mitogenome of the superfamily Noctuoidea reported so far.

Superfamily	Family	Subfamily	Species	Accession
Noctuoidea	Erebidae	Calpinae	*Oraesia emarginata*	MW648382*
			*Eudocima salaminia*	MW683337
			*Eudocima phalonia*	KY196412
		Catocalinae	*Catocala deuteronympha*	KJ432280
			*Grammodes geometrica*	KY888135
			*Parallelia stuposa*	MK262707
		Aganainae	*Asota plana lacteata*	KJ173908
		Herminiinae	*Hydrillodes lentalis*	MH013484
		Hypeninae	*Paragabara curvicornuta*	KT362742
		Arctiinae	*Hyphantria cunea*	GU592049
			*Vamuna virilis*	KJ364659
			*Amata formosae*	KC513737
			*Callimorpha dominula*	KP973953
			*Nyctemera arctata albofasciata*	KM244681
			*Paraona staudingeri*	KY827330
			*Spilosoma lubricipeda*	MT591568
			*Arctia plantaginis*	MW394229
			*Lemyra melli*	KP307017
			*Spilarctia subcarnea*	KT258909
			*Cyana* sp. MT-2014	KM244679
			*Eilema ussuricum*	MN696172
			*Aglaomorpha histrio*	KY800518
		Lymantriinae	*Lymantria dispar*	FJ617240
			*Lymantria dispar asiatica*	KY923067
			*Lymantria dispar japonica*	KY923060
			*Lymantria* sp. AN-2017	KY923068
			*Lymantria umbrosa*	KY923066
			*Lymantria sugii*	MT265380
			*Gynaephora minora*	KY688086
			*Gynaephora menyuanensis*	KC185412
			*Gynaephora jiuzhiensis*	KY688085
			*Gynaephora ruoergensis*	KY688083
			*Gynaephora qumalaiensis*	KJ507134
			*Gynaephora qinghaiensis*	KJ507133
			*Gynaephora aureata*	KY688084
			*Euproctis pseudoconspersa*	KJ716847
			*Euproctis similis*	KT258910
			*Euproctis cryptosticta*	KY996558
			*Euproctis seitzi*	MN916588
			*Somena scintillans*	MH051839
			*Lachana alpherakii*	KJ957168
			*Orgyia postica*	MW355619
			*Laelia suffusa*	MN908152
	Notodontidae	Phalerinae	*Phalera flavescens*	JF440342
		Thaumetopoeinae	*Ochrogaster lunifer*	AM946601
			*Clostera anachoreta*	KX108766
			*Clostera anastomosis*	MH286069
			*Thaumetopoea pityocampa*	MH286070
	Nolidae	Chloephorinae	*Gabala argentata*	KJ410747
			*Sinna extrema*	MG872330
		Risobinae	*Risoba prominens*	KJ396197
	Euteliidae	Euteliinae	*Eutelia adulatricoides*	KJ185131
		Stictopterinae	*Odontodes seranensis*	MW719565*
	Noctuidae		*Actinotia polyodon*	MW697903*
		Amphipyrinae	*Sesamia inferens*	JN039362
			*Spodoptera exigua*	JX316220
			*Spodoptera litura*	KF543065
			*Spodoptera frugiperda*	KU877172
			*Spodoptera littoralis*	MT816470
			*Spodoptera exempta*	MT906792
		Noctuinae	*Agrotis ipsilon*	KF163965
			*Agrotis segetum*	KC894725
			*Striacosta albicosta*	KM488268
		Xyleninae	*Athetis lepigone*	MF152842
			*Athetis pallidipennis*	MT040606
		Heliothinae	*Helicoverpa zea*	KJ930516
			*Helicoverpa punctigera*	KF977797
			*Helicoverpa armigera*	GU188273
			*Helicoverpa assulta*	MG437198
			*Helicoverpa gelotopoeon*	MG437199
			*Heliothis subflexa*	KT598688
			*Noctua pronuba*	KJ508057
		Acronictinae	*Acronicta psi*	KJ508060
		Hadeninae	*Mythimna separata*	KM099034
			*Mythimna pallidicosta*	MH027985
			*Mythimna loreyi*	MT506351
			*Leiometopon simyrides*	MW255962
			*Protegira songi*	KY379907
			*Mamestra configurata*	CM017530
			*Anarta trifolii*	MN715147
		Plusiinae	*Ctenoplusia agnata*	KC414791
			*Ctenoplusia limbirena*	KM244665
			*Ctenoplusia albostriata*	MN495624
			*Trichoplusia ni*	MK714850
			*Diachrysia nadeja*	MT916722
Hyblaeoidea	Hyblaeidae		*Hyblaea puera*	MW885970*
Pyraloidea	Crambidae	Crambinae	*Chilo suppressalis*	MK207057
			*Diatraea saccharalis*	FJ240227
		Pyraustinae	*Ostrinia furnacalis*	MN793323
			*Ostrinia nubilalis*	AF442957
Geometroidea	Geometridae	Ennominae	*Biston panterinaria*	JX406146
			*Phthonandria atrilineata*	EU569764
Bombycoidea	Sphingidae	Sphinginae	*Manduca sexta*	AY616435
	Saturniidae	Saturniinae	*Actias selene*	JX186589
			*Antheraea pernyi*	MT890592
	Bombycidae	Bombycinae	*Bombyx mori*	AF149768
			*Bombyx mandarina*	MK251840
Lasiocampoidea	Lasiocampidae		*Kunugia undans*	KX822016
		Pinarinae	*Trabala vishnou guttata*	KU884483
Outgroups			*Papilio polytes*	KM014701
			*Trogonoptera brookiana*	LT999986

* indicates the species that are used in this study.

All four species comprised a distinctive metazoan mitogenome composition of 13 protein-coding genes PCGs viz. ATPase subunits 6 and 8 (*atp6* and *atp8*), cytochrome c oxidase subunits 1–3 (*cox1, cox2* and *cox3*), NADH dehydrogenase subunits 1–6 (*nad1, nad2, nad3, nad4, nad5* and *nad6*), subunit 4L *nad* (*nad4l*) and cytochrome B (*cob*), 22 transfer RNA (*tRNA*) genes, two ribosomal genes (*rrnL* and *rrnS*) and a control region (A + T-rich region). Four of the thirteen PCGs (*nad1*, *nad4*, *nad4l*, and *nad5*) and eight tRNAs (*trnQ*, *trnC*, *trnY*, *trnF*, *trnH*, *trnP*, *trnL1* and *trnV*) and two rRNAs were encoded on the N-strand, whereas the other 23 genes (9 PCGs and 14 tRNAs) and the control-region (A + T-rich) were encoded on the J-strand (Table 2). All genes were organized in the same way without the rearrangement phenomenon.

**Table 2:** Details on gene organization of four newly determined Lepidoptera mitogenomes.

Gene/ strand	Position from	To	Anticodon	Start codon	Stop codon	Intergenic nucleotides
trnM/J	888/2512/2593/35	954/2580/2660/102	CAT			24/0/0/0
trnI/J	979/2581/2661/103	1046/2645/2727/167	GAT			-3/3/-3/-3
trnQ/N	1044/2643/2725/165	1112/2711/2793/233	TTG			63/56/57/48
nad2/J	1176/2768/2851/282	2189/3781/3864/1295		ATT/ATT/ATT/ATT	TAA/TAA/TAA/TAA	11/0/4
trnW/J	2201/3780/3869/1295	2270/3847/3938/1360	TCA			-8/-7/-8/-8
trnC/N	2263/3840/3931/1353	2334/3904/3997/1417	GCA			34/11/13/0
trnY/N	2369/3916/4011/1418	2433/3981/4077/1484	GTA			27/8/15/6
cox1/J	2461/3990/4093/1491	3996/5528/5626/3022		CGA/CGA/TTG/CGA	TAA/TAA/T/T	-5/-4/0/-1
trnL2/J	3992/5524/5627/3022	4058/5590/5693/3088	TAA			0/0/0/0
cox2/J	4059/5591/5694/3089	4734/6272/6375/3773		ATG/ATT/ATG/ATG	T/T/T/T	6/0/0/-2
trnK/J	4741/6273/6376/3771	4811/6343/6446/3841	CTT			12/3/-1/-1
trnD/J	4824/6347/6446/3841	4890/6414/6515/3907	GTC			0/0/0/0
atp8/J	4891/6415/6516/3908	5055/6576/6677/4072		ATA/ATT/ATT/ATT	TAA/TAA/TAA/TAA	-7/-7/-7/-7
atp6/J	5049/6570/6671/4066	5726/7247/7348/4740		ATG/ATG/ATG/ATG	TAA/TAA/TAA/TAA	-1/0/-1/-1
cox3/J	5726/7247/7348/4740	6514/8035/8136/5528		ATG/ATG/ATG/ATG	TAA/TAA/TAA/TAA	3/2/10/2
rnG/J	6518/8038/8147/5531	6584/8012/8212/5596	TCC			0/0/0/0
nad3/J	6585/8103/8213/5597	6938/8456/8566/5950		ATT/ATT/ATT/ATT	TAA/TAA/TAA/TAA	41/0/16/6
trnA/J	6980/8456/8583/5957	7045/8522/8649/6022	TGC			44/0/0/-1
trnR/J	7090/8526/8650/6022	7156/8591/8714/6087	TCG			21/11/-1/0
trnN/J	7178/8603/8714/6088	7243/8668/8779/6154	GTT			30/5/1/0
trnS1/J	7274/8674/8781/6155	7339/8739/8846/6220	GCT			9/0/3/19
trnE/J	7349/8740/8850/6240	7414/8806/8915/6306	TTC			15/2/4/-1
trnF/N	7430/8809/8920/6305	7495/8876/8986/6371	GAA			18/0/1/5
nad5/N	7514/8877/8988/6377	9244/10,622/10,733/8103		ATA/ATG/ATA/ATT	TTA/TAA/TAA/T	0/0/0/0
trnH/N	9245/10,623/10,734/8104	9311/10,689/10,799/8170	GTG			3/3/3/0
nad4/N	9315/10,693/10,803/8171	10,650/12,028/12,138/9511		ATG/ATG/ATG/ATG	T/T/T/TAA	15/20/51/6
nad4l/N	10,666/12,049/12,190/9518	10,953/12,339/12,477/9811		ATG/ATG/ATG/ATG	TAA/TAA/TAA/TAA	16/11/2/9
trnT/J	10,970/12,351/12,480/9821	11,035/12,416/12,544/9885	TGT			0/0/0/0
trnP/N	11,036/12,417/12,545/9886	11,100/12,481/12,610/9950	TGG			7/7/5/11
nad6/J	11,108/12,489/12,616/9962	11,631/13,022/13,149/10,495		ATA/ATG/ATT/ATG	T/TAA/TAA/TAA	18/11/13/24
cob/J	111,650/13,034/13,163/10,520	12,801/14,182/14,314/11,668		ATG/ATG/ATG/ATG	TAA/TAA/TAA/TAA	-1/2/-1/6
trnS2/J	12,801/14,185/14,314/11,675	12,868/14,250/14,380/11,742	TGA			44/18/21/16
nad1/N	12,913/14,269/14,402/11,759	13,851/15,207/15,340/12,694		ATT/ATG/ATG/ATG	TAA/ATT/TAA/TAG	0/1/0/1
trnL1/N	13,852/15,209/15,341/12,696	13,919/15,276/15,408/12,763	TAG			44/0/0/17
rrnL/N	13,964/1/20/12,781	15,280/1371/1356/14,056				32/-45/34/26
trnV/N	15,313/1327/1391/14,083	15,377/1393/1455/14,151	TAC			-1/0/0/0/-1
rrnS/N	15,377/1394/1456/14,152	16,114/2170/2236/14,913				0/0/0/-1
control region	601/2270/2249/14,912	862/2465/2592/15,350				

The order of the four species in the table is as follows: *Oraesia emarginata/Actinotia polyodon/ Odontodes seranensis/Hyblaea puera*. J and N refer to the majority and minority strand respectively.

The nucleotide compositions of the four moth mitogenomes had a high A + T bias: 79.72% in *O. emarginata*, 81.69% in *A. polyodon*, 81.09% in *O. seranensis*, and 81.21% in *H. puera*. Among the 88 Noctuoid species for which mtDNA data was available, the lowest A + T content was 77.83% in *O. lunifer*, while the highest A + T content was 81.69% in *Gabala argentata*. The mitogenome of *A. polyodon* was also highest among the known Noctuoid mitogenomes. All four mitogenomes showed a negative AT-skew on the majority strand and negative GC-skew as it occurred mostly among other Noctuoid mitogenomes. The AT and GC skew values on the majority strand of the four moths’ species are *O. emarginata* (−0.002 and −0.200), *A. polyodon* (−0.008 and −0.167), *O. seranensis* (−0.023 and −0.196), and *H. puera* (−0.000 and −0.178) (Supplementary Table 1). Similar patterns of nucleotide negative skew have also been found in the mitogenomes of other Noctuoid taxa^36,45,46,49^.

### Protein-coding genes and codon usage

The total lengths of the 13 PCGs of *O. emarginata*, *A. polyodon*, *O. seranensis* and *H. puera* were 11, 182 bp, 11, 213 bp, 11, 208 bp, and 11, 195 bp accounting for 67.08%, 73.06%, 72.68%, and 72.93% of the mitogenomes respectively. The locations and orientations of the 13 PCGs within the four mitogenomes were identical to those of most Noctuoid species. The nucleotide PCGs translated into 3716–3725 amino acid-coding codons, excluding the stop codons. Similar to the PCGs *nad5* and *atp8* were observed to be the largest (1727–1746 bp) and smallest (162–165 bp) genes, respectively. The majority of PCGs stringently started with an ATN (ATG/ATT/ATA) start codon, except *cox1* gene which started with CGA in *O. emarginata, A. polyodon* and *H. puera* and with TTG in *O. seranensis* (Table 1). The majority of PCGs terminated with a complete and canonical stop codon (TAA/TAG) except in *O. emarginata* where gene *nad5* terminated with TTA. The genes *cox2, nad4* (*O. emarginata, A. polyodon*), *cox1, cox2, nad4* (*O. seranensis*), *cox1, cox2,* and *nad5* (*H. puera*) were found to have a truncated termination codon (T) and it might be altered by post-transcriptional polyadenylation. The presence of an incomplete stop codon was also a common phenomenon in metazoan mitochondrial genes (Sheffield et al*.* 2010). The average A + T contents of the 13 PCGs within the four mitogenomes ranged from 78.85 to 80.36% (Supplementary Table 1).

**Figure 2:**
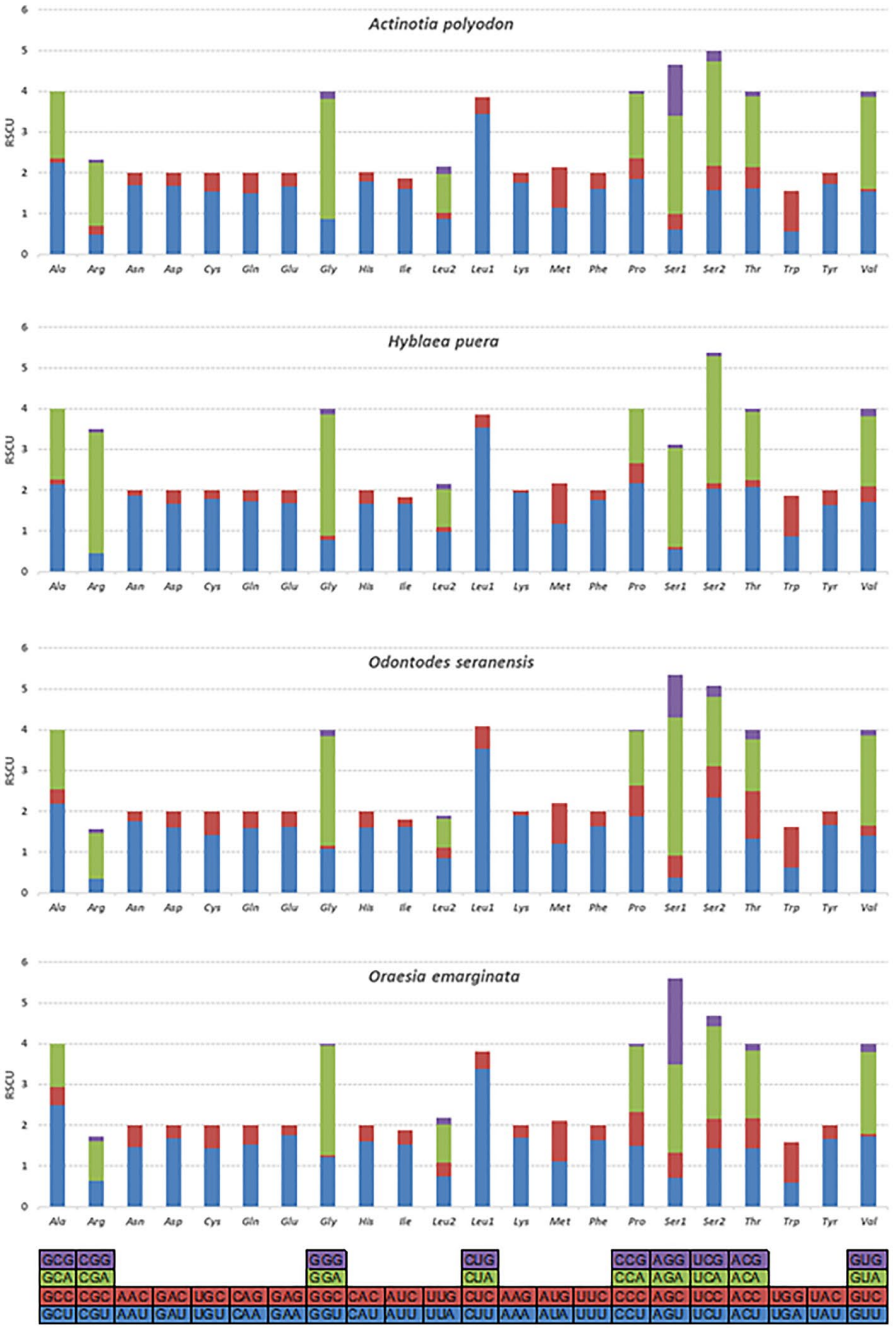
Relative Synonymous Codon Usage (RSCU) in Noctuoidea PCGs. The species name represents the superfamilies Noctuoidea & Hyblaeoidea. RSCU of *Actinotia polyodon,* RSCU of *Odontodes seranensis,* RSCU of *Oraesia emarginata* and RSCU of *Hyblaea puera.*

Relative synonymous codon usage (RSCU) was calculated in the mitogenomes of four lepidopterans (Fig. 2 and Supplementary Table 2). The most frequently utilized codons were almost similar within the four Noctuoid species. UUU (Phe), UUA (Leu), AUU (Ile), AAU (Asn) and AAA (Lys) were the most consistently used codons (> 232) within the PCGs of the four mitogenomes; however, GUG (V), ACG (Thr), CCG (Pro), GCC (Ala), and UGC (Cys) were the smallest used codons (< 10). We found average relative synonymous codons of 3737 (*A. polyodon*)*,* 3736 (*O. seranensis*), 3 727 (*O. emarginata*) and 3731 (*H. puera*), not including stop codons, that were predicted for codon usage of all the four mitogenomes.

### Overlapping and intergenic spacer regions

The intergenic sequences and overlapping regions were analyzed. The small intergenic spacers (IGS) were seen ranging in size 1–63 bp, and totalling 174 bp in *A. polyodon*, 256 bp in *O. seranensis*, 527 bp in *O. emarginata* and 262 bp in *H. puera* (Table 2). The longest intergenic spacer was located between *tRNA*^*Gln*^ and *nad2* with the length of 48 bp in *H. puera*, 56 bp in *A. polyodon*, 57 bp in *O. seranensis*, and 63 bp in *O. emarginata*.

In addition, the overlapping regions were also analyzed. The numbers of overlapping regions in four Noctuoid moths were mostly inconstant from 1 to 45 bp. However, the longest overlapping region was located between *rrnL* and *rRNA*^*Val*^, with a length of 45 bp in *A. polyodon* (Table 2).

### Transfer RNA genes (tRNA)

The mitogenomes of *H. puera*, *A. polyodon*, *O. seranensis*, *O. emarginata* had 22 tRNA genes (Table 2). The total lengths of the 22 tRNA genes were 1479 bp (*O. emarginata*), 1472 bp (*A. polyodon*), 1476 bp (*O. seranensis*) and 1471 bp (*H. puera*); however individual tRNA genes typically ranged from 65 to 71 bp, among which, eight tRNAs were encoded on the N-strand and the remaining 14 were encoded on the J-strand. The putative secondary structures of tRNA genes recognized in these Noctuoid mitogenomes are given in Supplementary Figs. 1–4. All the predicted tRNAs revealed the typical putative secondary structure except for *trnS1* (AGN) where dihydrouridine (DHU) arm lacked and formed a simple loop which has been found in many lepidopterans^22,30,39,40^. The lack of dihydrouridine (DHU) arm in *trnS1* (AGN) was observed in all species, while TΨC arm disappeared only in *trnE* of *O. emarginata*. In addition, TΨC loop was seen lacking in *trnY* and *trnF* of *O. emarginata*, and *trnI, trnT, trnF* of *H. puera*. Several mismatching base pairs occurred in tRNA clover-leaf secondary structures in all four lepidopteran mitogenomes. A total of 16 mismatches of 9 U-G and 7 G-U wobble pairs were noticed in 13 tRNA genes of *A. polyodon*; 18 mismatches (1 U-U and 9 U-G) and 8 G-U wobble pairs were detected in 15 tRNA genes of *O. seranensis*; 20 mismatches (1 A-A, 3 U-U and 10 U-G) and 6 G-U wobble pairs were observed in 13 tRNA genes of *H. puera*; 18 mismatches (1 U-U and 10 U-G) and 7 G-U wobble pairs were observed in 14 tRNA genes of *O. emarginata*.

### Ribosomal RNA genes

Two rRNA genes (*rrnL* and *rrnS*) are extremely conserved in Noctuoid mitogenomes, and each of the four mitogenomes contained these two rRNA genes. *rrnL* gene lengths were 1370 bp for *A. polyodon*, 1336 bp for *O. seranensis*, 1276 bp for *H. puera* and 1317 bp for *O. emarginata*; whereas *rrnS* were 777, 781, 762, and 738 bp (Supplementary Table 1). The rRNA genes of currently sequenced mitogenomes displayed a negative AT skew (−0.007 to −0.036) and GC skew (−0. 356 to −0. 483). *rrnL* gene was located between *trnL1* and *trnV*, and *rrnS* was located between *trnV* and the control region (Table 2).

### The A + T-rich region

**Figure 3:**
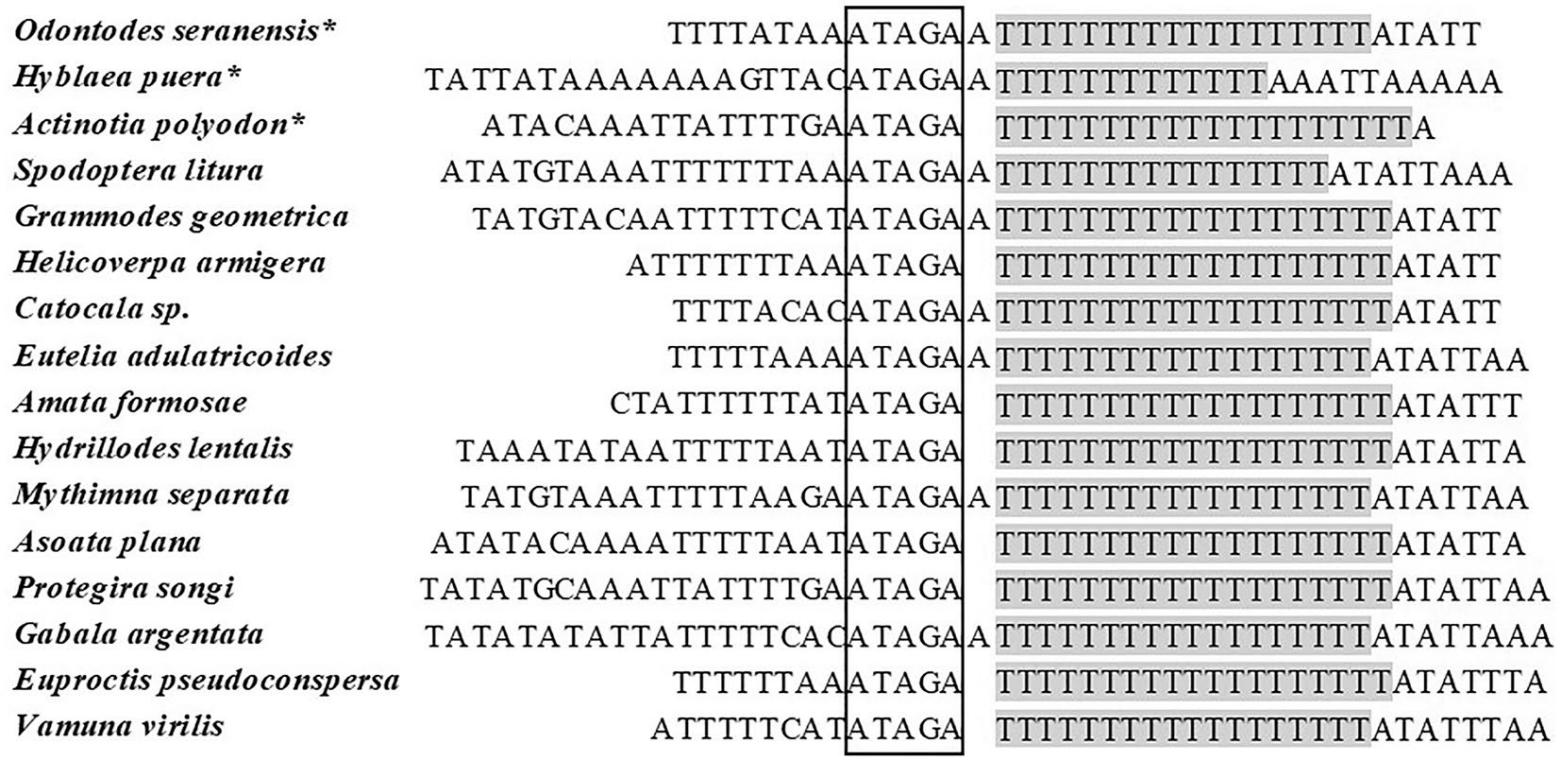
Alignment of initiation site for A + T-rich region of 16 species completely sequenced lepidopteran mitogenomes. The boxed nucleotides indicate the conserved motif ATAGA and the shaded nucleotides indicate poly-T stretch. * newly sequenced mitogenomes presented in this study.

The A + T-rich regions of *O. emarginata, A. polyodon*, *O. seranensis*, and *H. puera* were 287, 259, 343 and 439 bp in size respectively, all positioned between the *rrnS* and *tRNA*^*Met*^ (Table 2). The A + T content of these regions was 93.38%, 94.72%, 95.35% and 97.25%, respectively (Supplementary Table 1). The A + T-rich regions exhibited negative AT and GC-skew values. The conserved structure that connected the motif “ATAGA + poly-T stretch” was located downstream of the *rrnS* gene in the A + T-rich region of *H. puera*, *A. polyodon* and *O. seranensis*, which was not observed in *O. emarginata* (Fig. 3). We found that the motif ‘ATAGA’, might be the origin of light-strand replication^51^, directly connecting to the poly-T stretch in *A. polyodon* instead of (A)n which connected to the poly-T structure in *H. puera* and *O. seranensis*. Multiple tandem repeats are naturally existing in the A + T-rich region of most lepidopteran insects. We detected the presence of tandem repeats in the mitochondrial A + T-rich region in *A. polyodon*, *O. seranensis* and *H. puera*, but not in *O. emarginata*. The A + T-rich region of *H. puera* consisted of three tandem repeats each of size 128 bp, 113 bp and 108 bp. Only one tandem repeat (103 bp) was found in the A + T-rich region of *A. polyodon*. In *O. seranensis*, the A + T-rich region consisted of two tandem repeats (57 bp and 47 bp). However, the tandem repeat was not observed in the A + T-rich region of *O. emarginata*; similarly, the tandem was also not present in *Dysgonia stuposa*^32^.

**Figure 4:**
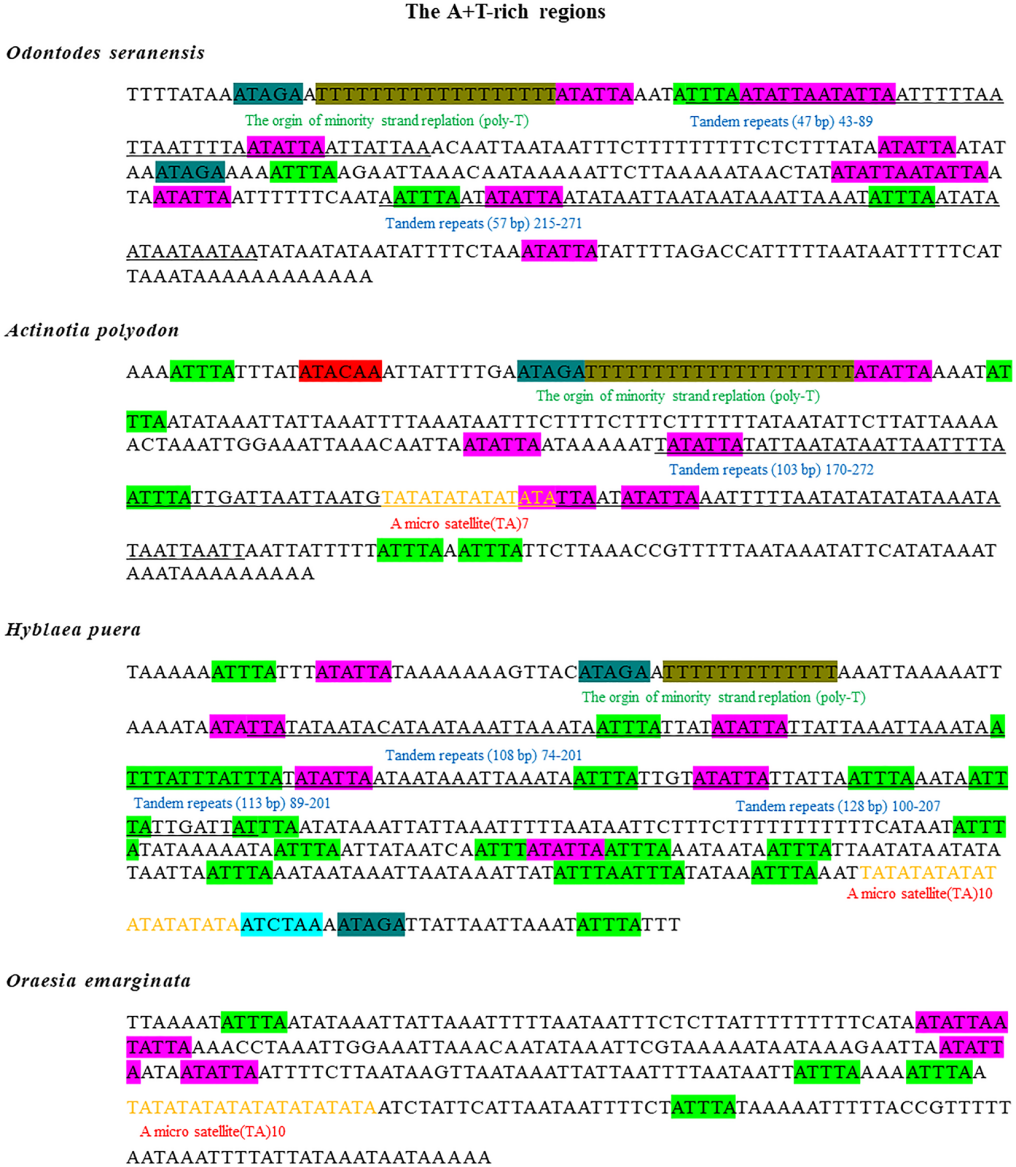
Motifs and microsatellites found in the A + T-rich region of *Odontodes seranensis, Actinotia polyodon, Hyblaea puera* and *Oraesia emarginata*. These are indicated by specific colours and highlights. Motifs (ATAGA) are shown in dark blue high lights. Poly-T stretch are shown in darker gold accent highlights. Microsatellites (ATATTA) are shown in pink highlights. Microsatellites (ATTTA) are shown in green highlight. All tandem repeats are underlined. (ATACAA) block is shown in light blue highlight. Microsatellite (TA)10 and (TA)7 are shown in yellow colour.

Additionally, two dinucleotide microsatellites and three motifs were detected in *H. puera*, referred to as (TA)_10_, (TA)_3_ in repeat 5, motif (ATAGA)_2_, (ATTTA)_16_, and TAATAATAA. In *A. polyodon,* one dinucleotide microsatellite (TA)_7_ and three motifs (ATTTA)_5_, (ATAGA)_1_ and TAATAATAA were also observed. One dinucleotide (TA)_3_, one trinucleotide (TAATAATAA)_2_ microsatellites, and three motifs (ATAGA)_2_, (ATTTA)_4_ and (ATATTA)_10_ were found in *O. seranensis*. Similarly, one dinucleotide microsatellite (TA)_10_ and two motifs (ATTTA)_4_ and (ATATTA)_3_ were found in *O. emarginata*. Furthermore, the ‘ATCTAA’ block in *H. puera* upstream of the origin of light-strand replication was different from the ‘ATACAA’ block in *A. polyodon* (Fig. 4).

### Phylogenetic relationships

**Figure 5:**
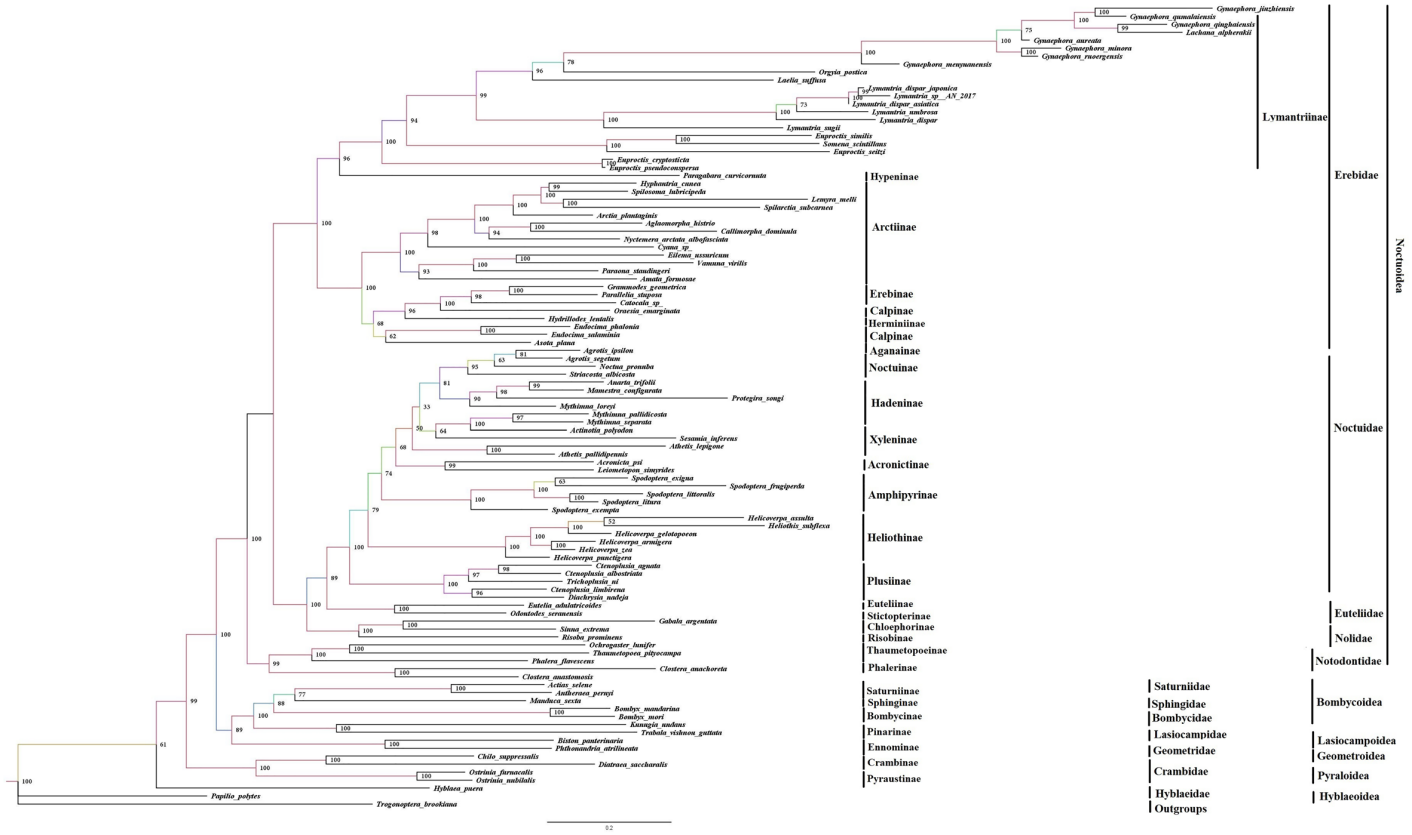
Phylogenetic tree of superfamily Noctuoidea moths using IQ-TREE. The phylogeny was reconstructed using 13 PCGs and two rRNA of the 90 species with maximum likelihood (ML) method (1000 replications). The species *Papilo polytes* and *Trogonoptera brookiana* mitogenomes were used as outgroups.

**Figure 6:**
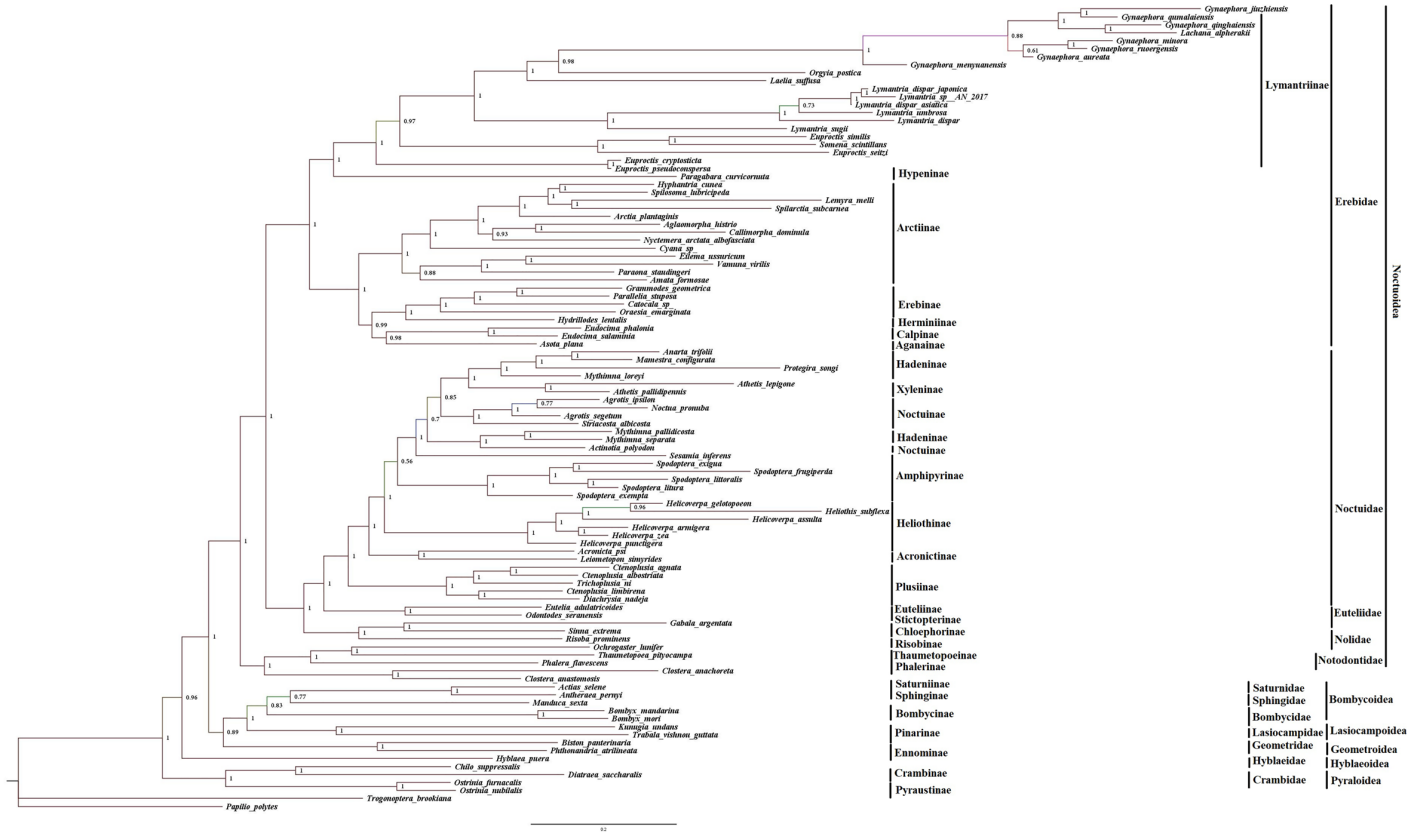
Phylogenetic tree of superfamily Noctuoidea moths using MrBayes. The phylogeny was reconstructed using 13 PCGs and two rRNA of the 90 species with Bayesian Inference. Posterior probability values lower than 50 were not shown.

We performed the phylogenetic study on the mitogenomes of 99 lepidopteran species representing five Noctuoid families (Erebidae, Euteliidae, Noctuidae, Nolidae, Notodontidae), one Hyblaeoid (Hyblaeidae), one Pyraloid (Crambidae), one Geometroid (Geometridae), three Bombycoid (Sphingidae, Saturniidae, Bombycidae), and one Lasiocampoid (Lasiocampidae) with two outgroup species (*Papilio polytes* and *Trogonoptera brookiana*) using the Maximum likelihood (ML) method and Bayesian inference (BI). The analyses were conducted on the dataset 13 PCGs + two rRNAs of the mitochondrial genomes which acquired similar tree topology (Figs. 5 and 6). We obtained the concatenated amino acid sequences to reconstruct the phylogenetic relationships (Figs. 5 and 6). The topology of the families based on mitogenomes in this study was consistent with the previous morphological and molecular studies^11,12,32,52–54^.

The phylogenetic trees consisted of 6 clades corresponding to 12 major lepidopteran families (Figs. 5 and 6). The family Erebidae formed a major clade including 43 species with high bootstrap proportion and posterior probability (BP ≥ 100; PP: 1). This clade is further comprised of two subclades; subclade I with strongly supported values (BP ≥ 100; PP: 1) involving five subfamilies; Arctiinae Erebinae, Calpinae, Herminiiinae, and Aganainae. The type species *Oraesia emarginata* belonging to the tribe Calpini was closely related to the *Catocala* sp., *Grammodes geometrica,* and *Parallelia stuposa* with well-supported values (BP ≥ 100; PP: 1); subclade II comprised of 22 species belonging to the subfamilies Lymantriinae and Hypeninae with high nodal support (BP ≥ 96; PP: 1), out of which a single species *Paragabara curvicornuta* belonging to the subfamily Hypeninae was clustered separately in the same clade. The subfamily Arctiinae is closely related to the subfamily Erebinae rather than Lymantriinae. The Erebidae clade showed the relationship as; ((Aganainae + Calpinae + Herminiinae + Erebinae + (Arctiinae + (Hypeninae + Lymantriinae)))).

The newly sequenced species *O. seranensis* and *Eutelia adulatricoides* clustered into a single clade with high bootstrap proportion (BP ≥ 100; PP: 1). These species belonged to the family Euteliidae and strongly supported a monophyletic group. This branch consisted of two subfamilies, Stictopterinae and Euteliinae. Formerly the Euteliinae and Stictopterinae were treated as separate subfamilies of Noctuidae^55–57^. Later these two were placed as subfamilies of Erebidae^53^. Afterwards, the position of subfamily Euteliinae was raised to the family level and Stictopterinae was placed into the subfamily of Euteliidae based on molecular study^18^. The present observation was well supported by the molecular study of Zahiri et al.^18^. The recently reconstituted family Nolidae with species *Gabala argentata, Sinna extrema* and *Risoba prominens* clustered in a single clade with high bootstrap proportion (BP ≥ 100; PP: 1) and observed more closely related to the clade (Euteliidae + Noctuidae), instead of Erebidae as proposed by Zahiri et al.^58^.

The target species *A. polyodon* and thirty-one species belonging to the family Noctuidae were clustered into single branches with high nodal support values (BP ≥ 100; PP: 1). The species *A. polyodon* is clustered with Hadeninae clade with a high support value (BP ≥ 100; PP: 1). This phylogenetic analysis showed the main topology: ((Plusiinae + (Heliothinae + (Amphipyrinae + (Acronictinae + (Xyleninae + ((Hadeninae + Noctuinae)))))))).

Notodontiidae was strongly supported as a monophyletic group (BP ≥ 99; PP: 1). The clade consisted of two subfamilies Phalerinae (*Phalera flavescens*), and Thaumetopoeinae (*Ochrogaster lunifer, Clostera anachoreta, Clostera anastomosis*, and *Thaumetopoea pityocampa*).

In the past decade, a number of studies have explored the molecular phylogenetic relationships among the Noctuoidea species. Zahiri et al.^18^ proposed the following among these families: (Notodontidae + (Euteliidae + (Noctuidae + Erebidae + Nolidae))). In comparison with this, Yang et al.^12^ published different study in which the following assemblage was proposed: (Notodontidae + (Erebidae + Nolidae + Euteliidae + Noctuidae))). All analyses clearly supported the monophyletic relationships of the 16 subfamilies within Noctuoidea (Figs. 5 and 6). The reformulated family Noctuidae clustered with the newly erected family Euteliidae. Our findings indicated that the branch of Noctuidae and Euteliidae was sister to the newly constituted family Nolidae. The family Erebidae was sister to the clade of (Nolidae + (Euteliidae + Noctuidae)). Family Notodontidae members formed as a single clade consisting of subfamilies, Phalerinae and Thaumetopoeinae. Notodontidae was the sister group to the other Noctuoid families. Our analysis revealed a topology within Noctuoidea as follows: (Notodontidae + (Erebidae + (Nolidae + (Euteliidae + Noctuidae)))). The superfamily Noctuoidea relationships further confirmed that Noctuoidea was a monophyletic group, which was also supported by many previous mitogenome phylogenies^12,32–34^.

In the present analyses, a total of 9 species were included belonging to the superfamilies Bombycoidea, Lasiocampoidea, and Geometroidea. The phylogenetic tree analyses showed that Saturniidae (*Actias selene* and *Antheraea pernyi*), Sphingidae (*Manduca sexta*), Bombycidae (*Bombyx mandarina* and *Bombyx mori*), Lasiocampidae (*Kunugia undans* and *Trabala vishnou guttata*) and Geometridae (*Biston panterinaria* and *Phthonandria atrilineata*) formed a clade with high nodal support values (BP≥89; PP: 0.89), this is consistent with earlier molecular study^59^. The tree topologies indicate that the relationships are ((Geometridae + (Lasiocampidae + (Bombycidae + ((Sphingidae + Saturniidae)))))). The phylogenetic analyses also revealed the relationships in the superfamilies Bombycoidea, Geometroidea, Lasiocampoidea, and Noctuoidea with strongly supported values (BP≥100; PP: 1). This relationship is the resemblance to the novel Lepidoptera classification revised by van Nieukerken et al.^11^ and the superfamilies are designated as the Macroheterocera clade.

Four species belonging to the family Crambidae formed a separate clade (BP ≥ 99; PP: 1) which was placed sister to the family Hyblaeidae and both families belong to the clade Obtectomera^11^. The present analysis is analogous to the molecular analysis by Twort et al.^44^ which also showed that *Hyblaea* is sister to Pyraloidea. The newly sequenced species *H. puera* is separately clustered with moderate support in ML analysis and high support in BI analysis (BP ≥ 61; PP: 0.96). This species was earlier classified under the family Noctuidae (Hampson, 1894). During the same year, the family Hyblaeidae was placed under the superfamily Pyraloidea based on the morphology characters^60^. Afterward, it got its own superfamily rank Hyblaeoidea and was placed under the Obtectomera clade^11^. In the present study, the species *H. puera* deviated from the Noctuoidea member groups. This mitogenome study is well supported by morphological^11^ and molecular studies^18^. Unfortunately, the presence of only one mitogenome of Hyblaeidae restricted the discussion of its relationships; more species need to be added for a meaningful inference.

## Conclusion

The complete mitochondrial genome sequences of *O. emarginata, O. seranensis, A. polyodon* and *H. puera* were successfully determined. The mitogenomes of these fourmoth species were all double-stranded single-circular molecules with similar gene arrangements (Fig. 1). The overall genomic characteristics (gene order, gene size, base composition, PCG codon usage, and tRNA cloverleaf structure) of the lepidopteran mitogenomes were typically constant with those of reported Lepidoptera mitogenomes. The longest intergenic spacer was present between *trnQ* and *nad2;* this was a unique feature in all sequenced species. Based on the phylogenetic analyses, the amino acid datasets supported the monophyly of Noctuoidea and its relationships (Notodontidae + (Erebidae + (Nolidae + (Euteliidae + Noctuidae)))). However, more mitochondrial genome samples need to be used to further resolve the relationships among the Noctuoidea.

## Materials and methods

### Sample collection and genomic DNA extraction

The samples of the four species, *O. emarginata* (11° 41′ 181″ N 76° 72′ 07″ E), *A. polyodon, O. seranensis* (10° 23′ 5367˝ N 77° 49′ 2933˝ E) and *H. puera* (10° 27′ 045˝ N 77° 53′ 3633˝ E) were collected from the Tamil Nadu part of Western Ghats. K. Sivasankaran identified all the species, which were preserved in absolute ethanol and stored at -80ºC until DNA isolation. The genomic DNA was extracted from thorax tissue of moths using *Quick-*DNA Tissue/Insect Microprep Kit (Cat No-D6016-HSN CODE-38220090, Zymo Research, USA) with the manufacturer’s protocol. The DNA samples and quality were checked using Nanodrop 1000 and confirmed with 1% agarose gel.

### Mitogenome sequencing

The quality-check passed samples were subjected further for the library preparation. In brief, 100 ng of DNA was subjected to prepare indexed library using Truseq Nano library preparation kit (Illumina #20,015,964). Final libraries were quantified using Qubit 4.0 fluorometer (Thermofisher #Q33238) using DNA HS assay kit (Thermofisher #Q32851) following manufacturer’s protocol. To identify the insert size of the library, we queried it on Tapestation 4150 (Agilent) utilizing highly sensitive D1000 screen tapes (Agilent # 5067–5582) following manufacturers’ protocol. The next-generation sequencing was performed by Molsys Scientific Pvt. Ltd (Bangalore, India). Finally, NOVASEQ 6000 platform (Illumina, San Diego, California USA) was used to sequence 151 bp read lengths about 4 GB in size.

### Sequence assembly and annotation

The raw sequences were assembled using the NOVOPLASTY Ver 4.2 (https://github.com/ndierckx/NOVOPlasty)^61^. The sequences’ annotations were executed using MITOS2 (http://mitos2.bioinf.uni-leipzig.de/index.py)^62^ using the genetic code for invertebrate mitogenomes. The sequences were also annotated and verified for accurate lengths of the 13 protein-coding genes using CHLOROBOX-GeSeq-Annotation of Organellar Genomes (https://chlorobox.mpimp-golm.mpg.de/geseq.html)^63^. The composition skewness was calculated using the formula: AT skew = [A − T]/[A + T]; similarly, GCskew = [G − C]/[G + C] (https://en.vectorbuilder.Com/tool/gc-content-calculator). The tRNA genes and their cloverleaf structures were predicted with MITOS2 software and analyzed by comparison with the nucleotide sequence of other lepidopteran tRNA sequences. Tandem repeats at the A + T-rich region were identified using the online Tandem Repeats Finder tool (http://tandem.bu.edu/trf/trf.html). Relative Synonymous Codon Usage (RSCU) of PCGs was determined using MEGA X^64^. The circular maps of the four complete mitogenomes were drawn using the OGDRAW-Draw Organelle Genome Maps (https://chlorobox.mpimp-golm.mpg.de/OGDraw.html)^65^.

### Phylogenetic analyses

A total of 90 species (4 newly sequenced in this study, 86 available from GenBank) representing 7 families of Lepidoptera^11^ were used to reconstruct the phylogenetic relationships among them. The ingroup consisted of 43 species of Erebidae, 2 species of Euteliidae, 32 species of Noctuidae, 3 species of Nolidae, 5 species of Notodontidae, 1 species of Hyblaeidae, and 4 species of Crambidae. Species *Papilio polytes* and *Trogonoptera brookiana* mitogenomes were selected as outgroups (Table 1).

The amino acid sequences of 13 protein-coding genes and two rRNA genes were used in phylogenetic analysis. We used MAFFT to align and concatenate each of the 13 PCGs and rRNAs genes. Further the concatenated amino acid sequences from the 13 PCGs and rRNA genes were used for reconstructing the phylogenetic tree, which was performed using the Model-based Maximum Likelihood method using the IQ-TREE in PhyloSuite V1.2.2 program https://github.com/dongzhang0725/PhyloSuite^66^. The appropriate model General Reversible mitochondrial (mtREV) Gamma distributed with invariant sites (G + I) was used to infer the phylogenetic relationships based on 5000 bootstraps of ultrafast replicates.

The analysis of Bayesian inference (BI) was conducted for the dataset. The BI analysis was performed through the MrBayes 3.2.6 in PhyloSuite V1.2.2^66^ using the GTR + I + R model. Invgamma (+ I + G proportion invariable, remaining gamma rate variations across sites were presented and performed. The convergence of Markov Chain Monte Carlo (MCMC), which was observed by the average standard deviation of split frequencies, reached below 0.01. Four chains (three hot and one cold) were run with a dataset for one million generations with the tree being sampled every 1000 generations with a burn-in of 2500. FigTree v1.4.4 (http://tree.bio.ed.ac.uk/software/figtree/) was practised to visualise the phylogenetic tree.

## Supplementary Information

Supplementary Information 1.

Supplementary Information 2.

Supplementary Information 3.

Supplementary Information 4.

Supplementary Information 5.

Supplementary Information 6.
