# Supplementary figures and images for: Characterization of four mitochondrial genomes from superfamilies Noctuoidea and Hyblaeoidea with their phylogenetic implications
Source: Sci Rep. 2022 Nov 7;12:18926. doi: 10.1038/s41598-022-21502-y (PMC9640664; doi:10.1038/s41598-022-21502-y)

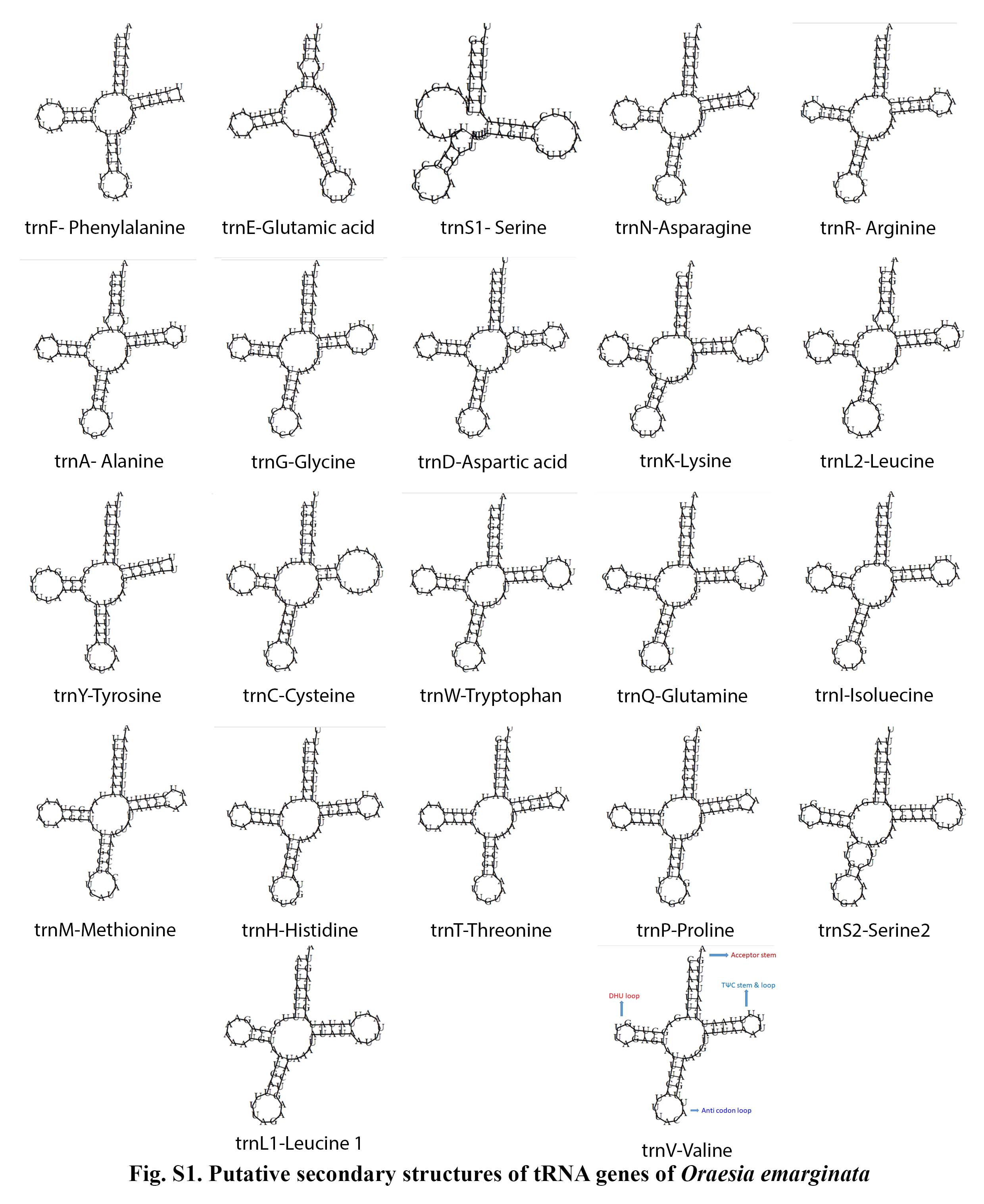

Supplement: Supplementary file 1 — Supplementary Information 1. [file 41598_2022_21502_MOESM1_ESM.jpg]

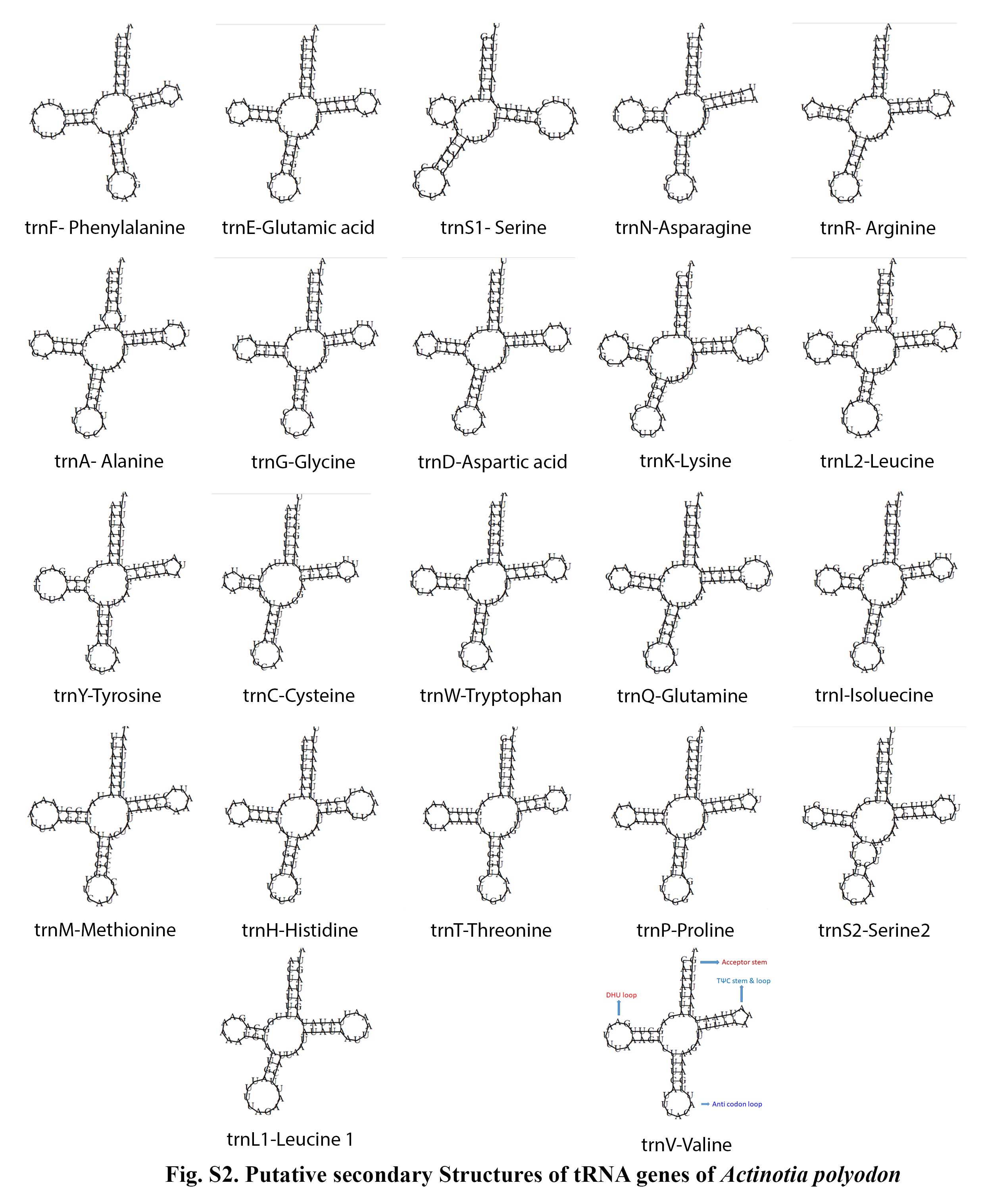

Supplement: Supplementary file 2 — Supplementary Information 2. [file 41598_2022_21502_MOESM2_ESM.jpg]

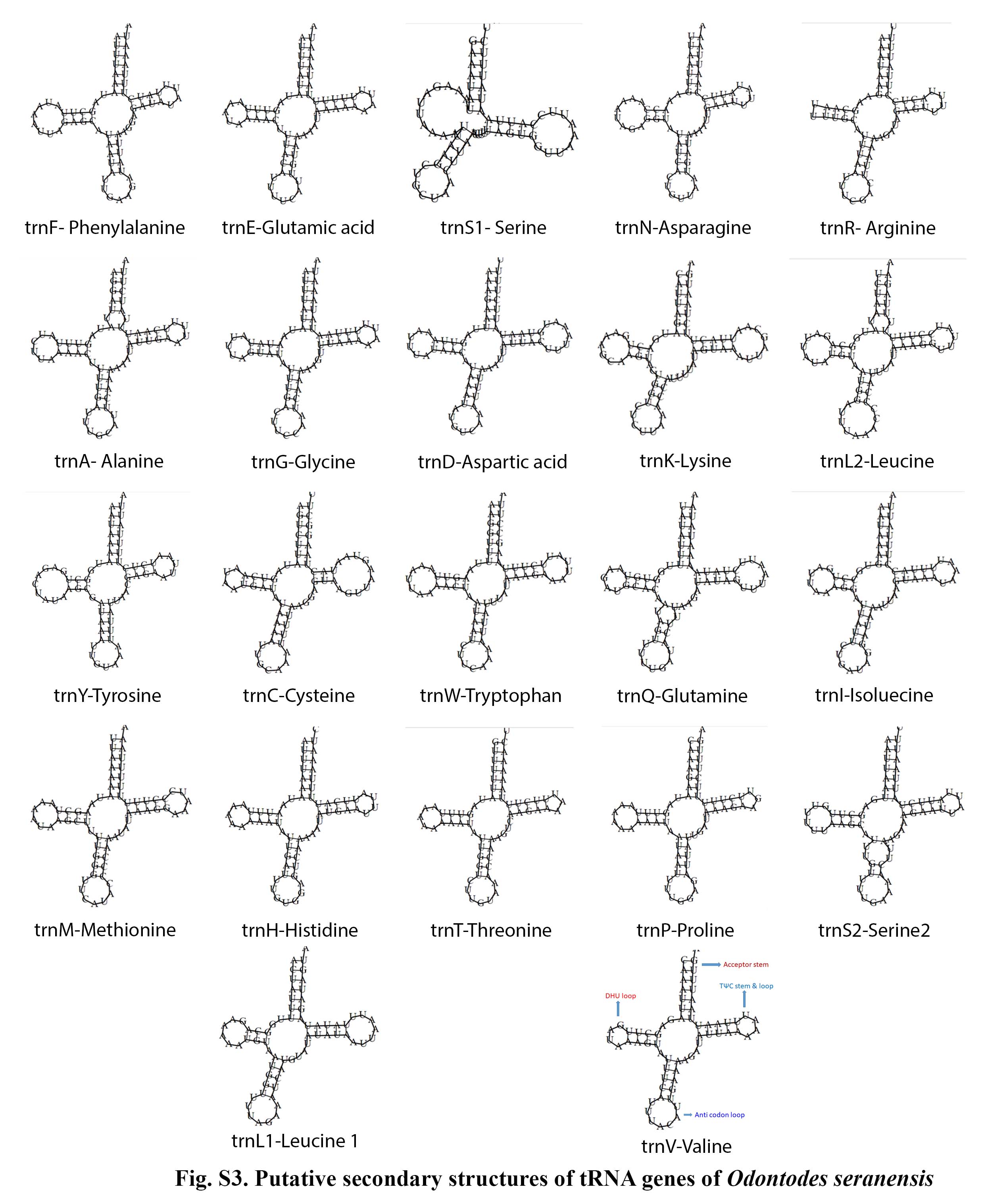

Supplement: Supplementary file 3 — Supplementary Information 3. [file 41598_2022_21502_MOESM3_ESM.jpg]

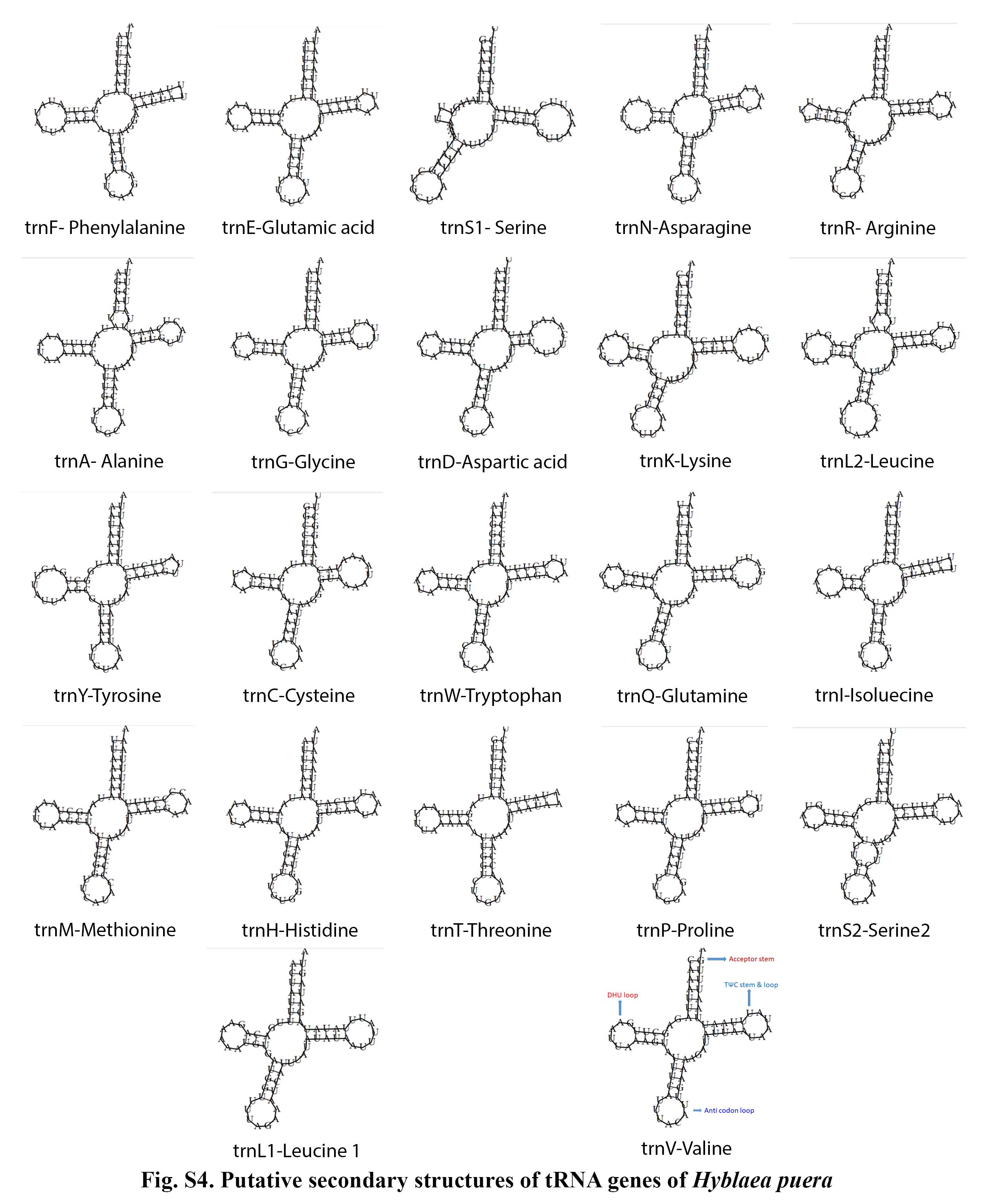

Supplement: Supplementary file 4 — Supplementary Information 4. [file 41598_2022_21502_MOESM4_ESM.jpg]
