# Supplementary material for: Characterization of four mitochondrial genomes from superfamilies Noctuoidea and Hyblaeoidea with their phylogenetic implications
Source: Sci Rep. 2022 Nov 7;12:18926. doi: 10.1038/s41598-022-21502-y (PMC9640664; doi:10.1038/s41598-022-21502-y)
Supplement: Supplementary file 5 — Supplementary Information 5. [file 41598_2022_21502_MOESM5_ESM.docx]

**Supplementary Table 1**. Nucleotide compositions and skewness in superfamily Noctuoidea mitogenomes

| **Species** | **Size (bp)** | **A %** | **G%** | **T%** | **C%** | **A+T%** | **AT skew** | **GC skew** |
| --- | --- | --- | --- | --- | --- | --- | --- | --- |
| *Odontodes seranensis* | 15,419 | 39.59 | 7.6 | 41.5 | 11.31 | 81.09 | -0.023 | -0.196 |
| *Hyblaea puera* | 15,350 | 40.59 | 7.73 | 40.61 | 11.08 | 81.21 | -0.000 | -0.178 |
| *Actinotia polyodon* | 15,347 | 40.48 | 7.62 | 41.21 | 10.69 | 81.69 | -0.008 | -0.167 |
| *Oraesia emarginata* | 16,668 | 39.78 | 8.11 | 39.94 | 12.18 | 79.72 | -0.002 | -0.200 |
| *Eudocima salaminia* | 15,597 | 42.02 | 9.39 | 38.41 | 10.16 | 80.44 | 0.044 | 0.039 |
| *E. phalonia* | 15,575 | 39.83 | 7.54 | 40.84 | 11.76 | 80.67 | -0.012 | -0.218 |
| *P. flavescens* | 15,659 | 40.07 | 7.86 | 40.80 | 11.25 | 80.87 | -0.009 | -0.177 |
| *O. lunifer* | 15,593 | 40.09 | 7.43 | 37.74 | 14.59 | 77.83 | 0.030 | -0.325 |
| *A. formosae* | 15,463 | 38.66 | 7.52 | 40.82 | 12.97 | 79.48 | -0.027 | -.0.267 |
| *Doa* sp. | 15,228 | 40.41 | 7.76 | 40.01 | 11.64 | 80.42 | 0.004 | -0.2 |
| *N. arctata albofasciata* | 15,431 | 39.27 | 7.34 | 40.37 | 11.66 | 79.64 | -0.013 | -0.227 |
| *Lymantria dispar* | 15,569 | 40.58 | 7.57 | 39.29 | 12.55 | 79.87 | 0.016 | -0.247 |
| *Lymantria* sp. AN-2017 | 15,651 | 39.46 | 12.42 | 40.72 | 7.39 | 80.18 | -0.015 | 0.253 |
| *L. umbrosa* | 15,635 | 39.39 | 12.52 | 40.58 | 7.49 | 79.97 | -0.014 | 0.251 |
| *Helicoverpa zea* | 15,343 | 40.59 | 7.58 | 40.40 | 11.41 | 80.99 | 0.002 | -0.020 |
| *H. gelotopoeon* | 15,229 | 40.35 | 7.59 | 40.44 | 11.60 | 80.80 | -0.001 | -0.208 |
| *H. armigera* | 15,347 | 40.54 | 7.68 | 40.43 | 11.33 | 80.97 | 0.001 | -0.192 |
| *H. cunea* | 15,481 | 40.57 | 7.55 | 39.81 | 12.05 | 80.38 | 0.009 | -0.229 |
| *H. puntigera* | 15,382 | 40.69 | 7.58 | 40.65 | 11.06 | 81.34 | 0.000 | -0.186 |
| *H. assulta* | 15,351 | 40.59 | 7.73 | 40.42 | 11.31 | 81.01 | 0.002 | -0.188 |
| *Heliothis subflexa* | 15,323 | 40.44 | 7.81 | 40.29 | 11.45 | 80.72 | 0.002 | -0.189 |
| *A. ipsilon* | 15,377 | 40.38 | 7.71 | 40.86 | 11.03 | 81.24 | -0.005 | -0.177 |
| *A. segetum* | 15,378 | 40.20 | 7.79 | 40.49 | 11.50 | 80.69 | -0.003 | -0.192 |
| *Spodoptera litura* | 15,374 | 41.03 | 7.60 | 40.00 | 11.36 | 81.03 | 0.012 | -0.198 |
| *S. frugiperda* | 16,346 | 40.38 | 7.97 | 40.35 | 11.29 | 80.73 | 0.000 | -0.172 |
| *S. exigua* | 15,365 | 40.87 | 7.67 | 40.05 | 11.38 | 80.92 | 0.010 | -0.194 |
| *C. agnata* | 15,261 | 39.57 | 7.70 | 41.51 | 11.19 | 81.08 | -0.030 | -0.184 |
| *C.limbirena* | 15,306 | 39.06 | 7.84 | 41.95 | 11.15 | 81.01 | -0.035 | -0.174 |
| *E. adulatricoides* | 15,360 | 40.20 | 7.80 | 40.65 | 11.32 | 80.85 | -0.005 | -0.184 |
| *S. inferens* | 15,413 | 40.06 | 7.61 | 40.17 | 12.15 | 80.23 | -0.001 | -0.229 |
| *N. pronuba* | 15,315 | 39.79 | 7.80 | 41.27 | 11.00 | 81.06 | -0.018 | -0.170 |
| *A. psi* | 15,350 | 40.89 | 7.82 | 38.19 | 12.92 | 79.08 | 0.034 | -0.168 |
| *Striacosta albicosta* | 15,553 | 40.14 | 7.87 | 39.18 | 12.79 | 79.32 | 0.012 | -0.238 |
| *Mythimna separata* | 15,329 | 40.02 | 7.66 | 40.98 | 11.33 | 81.00 | -0.011 | 0.193 |
| *M. pallidicosta* | 15,320 | 39.86 | 7.84 | 40.34 | 11.94 | 80.20 | -0.006 | -0.207 |
| *R. prominens* | 15,343 | 40.24 | 7.80 | 40.81 | 11.13 | 81.05 | -0.007 | -0.175 |
| *Catocala* *deuteronympha* | 15,671 | 39.68 | 7.26 | 41.43 | 11.61 | 81.11 | -0.021 | -0.230 |
| *Grammodes geometrica* | 15,728 | 40.10 | 7.61 | 40.38 | 11.89 | 80.49 | -0.003 | -0.219 |
| *Asota plana* | 15,416 | 40.08 | 7.49 | 40.26 | 12.16 | 80.34 | -0.002 | -0.237 |
| *Athetis lepidone* | 15,589 | 39.68 | 7.57 | 41.65 | 11.08 | 81.33 | -0.024 | -0.188 |
| *Hydrillodes lentalis* | 15,570 | 39.72 | 7.50 | 41.35 | 11.41 | 81.07 | -0.020 | -0.207 |
| *Protegira songi* | 15,410 | 40.10 | 7.85 | 40.11 | 11.93 | 80.21 | -8.08 | -0.20 |
| *Mamestra configurata* | 15,347 | 40.54 | 7.59 | 40.83 | 11.02 | 81.38 | -0.003 | -0.184 |
| *Paragabara curvicornuta* | 15,531 | 39.89 | 7.57 | 40.55 | 11.98 | 80.44 | -0.008 | -0.225 |
| *Gabala argentata* | 15,337 | 39.64 | 7.56 | 42.04 | 10.74 | 81.69 | -0.029 | -0.173 |
| *Gynaephora minora* | 15,801 | 40.96 | 6.76 | 40.51 | 11.75 | 81.48 | 0.005 | -0.269 |
| *G. menyuanensis* | 15,770 | 40.87 | 6.88 | 40.60 | 11.75 | 81.47 | 0.003 | -0.261 |
| *G. jiuzhiensis* | 15,859 | 40.91 | 6.72 | 40.63 | 11.73 | 81.54 | 0.003 | 0.271 |
| *G. aureata* | 15,761 | 40.94 | 6.77 | 40.51 | 11.76 | 81.45 | 0.005 | -0.269 |
| *G. ruoergensis* | 15,803 | 41.03 | 6.71 | 41.03 | 11.76 | 81.52 | 0.006 | -0.273 |
| *G. qumalaiensis* | 15,753 | 40.96 | 6.82 | 40.39 | 11.81 | 81.35 | 0.007 | -0.267 |
| *G. qinghaiensis* | 15,747 | 40.82 | 6.82 | 40.45 | 11.89 | 81.28 | 0.004 | -0.271 |
| *Euproctis similis* | 15,437 | 40.09 | 7.50 | 40.07 | 12.32 | 80.16 | 0.000 | -0.242 |
| *E. pseudoconspersa* | 15,461 | 40.41 | 7.60 | 39.51 | 12.46 | 79.93 | 0.011 | -0.242 |
| *E. cryptosticta* | 15,462 | 40.38 | 7.60 | 39.47 | 12.53 | 79.86 | 0.011 | -0.244 |
| *Somena scintillans* | 15,410 | 40.05 | 7.20 | 40.76 | 11.97 | 80.81 | -0.008 | -0.248 |
| *Lachana alpherakii* | 15,755 | 40.88 | 6.84 | 40.55 | 11.71 | 81.44 | 0.003 | -0.262 |
| *Vamuna virilis* | 15,417 | 40.17 | 7.55 | 40.21 | 12.05 | 80.39 | -0.000 | -0.229 |
| *Callimorpha dominula* | 15,496 | 40.07 | 7.58 | 40.94 | 11.39 | 81.02 | -0.010 | -0.200 |
| *Lemyra melli* | 15,418 | 39.38 | 8.26 | 39.28 | 13.06 | 78.66 | 0.001 | -0.224 |
| *Spilarctia alba* | 15,447 | 39.76 | 7.64 | 41.14 | 11.44 | 80.90 | -0.017 | -0.199 |
| *Spilarctia subcarnea* | 15,441 | 39.97 | 7.95 | 39.78 | 12.29 | 79.75 | 0.002 | -0.214 |
| *Cyana* sp. MT-2014 | 15,494 | 40.02 | 7.29 | 41.17 | 11.50 | 81.19 | -0.014 | -0.223 |
| *Eilema ussuricum* | 15,344 | 40.21 | 7.55 | 40.25 | 11.97 | 80.46 | -0.002 | -0.226 |
| *Aglaomorpha histrio* | 15,470 | 39.83 | 7.49 | 40.07 | 12.59 | 79.90 | -0.002 | -0.254 |
| *Clostera anachoreta* | 15,456 | 39.58 | 7.55 | 41.13 | 11.73 | 80.71 | -0.019 | -0.216 |
| *Clostera anastomosis* | 15,390 | 39.66 | 7.79 | 40.38 | 12.15 | 80.04 | -0.009 | -0.218 |
| **PCGs** |  |  |  |  |  |  |  |  |
| *Odontodes seranensis* | 11,208 | 39.13 | 8.39 | 40.47 | 12.01 | 79.6 | -0.016 | -0.177 |
| *Hyblaea puera* | 11,195 | 39.9 | 8.54 | 39.84 | 11.72 | 79.74 | 0.000 | -0.156 |
| *Actinotia polyodon* | 11,213 | 40.14 | 8.34 | 40.22 | 11.3 | 80.36 | -0.000 | -0.150 |
| *Oraesia emarginata* | 11,182 | 39.47 | 8.46 | 39.38 | 12.7 | 78.85 | 0.001 | -0.200 |
| *Eudocima salaminia* | 11,172 | 41.87 | 10.74 | 36.67 | 10.71 | 78.54 | 0.066 | 0.001 |
| *E. phalonia* | 10,809 | 38.90 | 8.55 | 39.49 | 13.04 | 78.39 | -0.007 | -0.207 |
| *P. flavescens* | 11,211 | 33.73 | 10.95 | 45.23 | 10.08 | 78.07 | -0.146 | -0.041 |
| *O. lunifer* | 11,266 | 32.47 | 12.08 | 43.26 | 12.19 | 75.73 | -0.142 | -0.004 |
| *H. cunea* | 11,205 | 33.59 | 8.10 | 45.00 | 10.42 | 79.59 | -0.145 | 0.027 |
| 1. *formosae* | 10,782 | 37.92 | 8.51 | 39.39 | 14.16 | 77.31 | -0.019 | 0.249 |
| *Doa* sp. | 10,747 | 39.70 | 8.73 | 38.55 | 12.60 | 78.65 | 0.009 | -0.181 |
| *N. arctata albofasciata* | 10,761 | 39.07 | 8.33 | 39.86 | 12.72 | 78.93 | -0.010 | -0.208 |
| *L. dispar* | 11,236 | 33.22 | 11.26 | 44.62 | 10.90 | 77.84 | -0.146 | 0.016 |
| *G. menyuanensis* | 11,228 | 34.63 | 10.18 | 45.15 | 10.04 | 79.78 | -0.132 | 0.007 |
| *H. armigera* | 10,791 | 39.79 | 8.64 | 39.20 | 12.38 | 78.95 | 0.006 | 0.177 |
| *H. puntigera* | 10,788 | 39.84 | 8.53 | 39.58 | 12.04 | 79.42 | 0.003 | 0.170 |
| *H. assulta* | 10,791 | 39.76 | 8.64 | 39.20 | 12.38 | 78.96 | 0.007 | -0.177 |
| *A. ipsilon* | 10,822 | 39.53 | 8.66 | 39.86 | 11.93 | 79.39 | -0.004 | -0.158 |
| *A. segetum* | 10,785 | 39.29 | 8.87 | 39.25 | 12.57 | 78.54 | 0.004 | 0.172 |
| *S. litura* | 11,206 | 40.30 | 8.38 | 39.26 | 12.06 | 79.56 | 0.012 | -0.180 |
| *S. exigua* | 10,766 | 40.08 | 8.69 | 38.92 | 12.28 | 79.00 | 0.014 | -0.171 |
| *C. agnata* | 10,782 | 38.81 | 8.58 | 40.60 | 11.99 | 79.41 | -0.022 | -0.165 |
| *C. limbirena* | 10,764 | 38.19 | 8.83 | 40.90 | 12.06 | 79.09 | -0.034 | -0.154 |
| *E. adulatricoides* | 10,800 | 39.25 | 8.82 | 39.74 | 12.18 | 78.99 | -0.006 | -0.159 |
| *S. inferens* | 10,905 | 39.35 | 8.50 | 38.96 | 13.17 | 78.31 | 0.005 | -0.215 |
| *N. pronuba* | 10,744 | 39.08 | 8.78 | 40.28 | 11.84 | 79.36 | -0.015 | -0.148 |
| *A. psi* | 10,725 | 40.22 | 8.86 | 36.69 | 14.20 | 76.91 | 0.045 | -0.231 |
| *Striacosta albicosta* | 11,210 | 39.46 | 8.79 | 37.99 | 13.76 | 77.46 | 0.019 | -0.220 |
| *M. separata* | 11,211 | 39.43 | 8.38 | 40.17 | 12.03 | 79.59 | -0.009 | -0.179 |
| *R. prominens* | 10,506 | 38.97 | 8.84 | 40.15 | 12.02 | 79.12 | -0.102 | -0.152 |
| *Catocala deuteronympha* | 10,884 | 38.80 | 8.23 | 40.28 | 12.66 | 79.08 | -0.187 | -0.212 |
| *Asota plana* | 10,626 | 39.18 | 8.59 | 38.78 | 13.43 | 77.96 | 0.005 | -0.219 |
| *P. curvicornata* | 11,196 | 39.29 | 8.42 | 39.40 | 12.87 | 78.69 | -0.001 | -0.20 |
| *P. songi* | 11,215 | 39.52 | 8.67 | 38.97 | 12.82 | 78.49 | 0.007 | -0.19 |
| *S. scintillans* | 11,201 | 39.46 | 8.04 | 39.38 | 13.11 | 78.84 | 0.001 | -0.23 |
| *S. subcarnea* | 11,214 | 39.73 | 8.58 | 38.71 | 12.96 | 78.44 | 0.01 | -0.20 |
| *S. frugiperda* | 11,197 | 39.81 | 8.45 | 39.53 | 11.94 | 79.34 | 0.006 | -0.17 |
| *V. virilis* | 11,203 | 39.64 | 8.39 | 38.92 | 13.03 | 78.56 | 0.008 | -0.21 |
| *A. lepigone* | 11,218 | 39.12 | 8.46 | 40.63 | 11.79 | 79.75 | -0.01 | -0.45 |
| *C. dominula* | 11,215 | 39.64 | 8.02 | 40.56 | 11.77 | 80.2 | -0.01 | -0.18 |
| *C. anachoreta* | 11,189 | 39.06 | 8.27 | 40.16 | 12.52 | 79.22 | -0.01 | -0.20 |
| *C. anastomosis* | 11,186 | 39.04 | 8.64 | 39.2 | 13.12 | 78.24 | -0.001 | -0.20 |
| *Cyana* sp*.* | 11,230 | 39.47 | 8.1 | 40.05 | 12.37 | 79.52 | -0.006 | -0.20 |
| *E. cryptosticta* | 11,187 | 39.64 | 8.45 | 38.26 | 13.66 | 77.9 | 0.017 | -0.23 |
| *E. psuedoconspersa* | 11.187 | 39.69 | 8.43 | 38.3 | 13.58 | 77.99 | 0.017 | -0.23 |
| *E. similis* | 11,202 | 39.5 | 8.37 | 38.83 | 13.29 | 78.33 | 0.008 | -0.22 |
| *G. argentata* | 11,203 | 39.05 | 8.29 | 41.27 | 11.38 | 80.32 | -0.02 | -0.15 |
| *G. geometrica* | 11,197 | 39.23 | 8.54 | 39.26 | 12.97 | 78.49 | 0 | 0 |
| *G. aureata* | 11,237 | 40.42 | 7.49 | 39.31 | 12.78 | 79.73 | 0.013 | -0.26 |
| *G. jiuzhiensis* | 11,246 | 40.41 | 7.51 | 39.39 | 12.68 | 79.80 | 0.012 | -0.25 |
| *H. gelotopoeon* | 11,209 | 39.87 | 8.30 | 39.57 | 12.27 | 79.44 | 0.003 | -0.193 |
| *G. minora* | 11,237 | 40.42 | 7.50 | 39.27 | 12.79 | 79.69 | 0.014 | -0.260 |
| *G. qinghaiensis* | 11,228 | 40.32 | 7.50 | 39.29 | 12.79 | 79.61 | 0.012 | -0.256 |
| *G. qumalensis* | 11,225 | 40.45 | 7.55 | 39.22 | 12.76 | 79.67 | 0.015 | -0.256 |
| *G. ruorgensis* | 11,237 | 40.47 | 7.44 | 39.28 | 12.78 | 79.75 | 0.014 | -0.263 |
| *H. lentalis* | 11,163 | 39.17 | 8.43 | 40.19 | 12.19 | 79.36 | -0.012 | -0.181 |
| *L. alpherakii* | 11,122 | 40.63 | 7.70 | 39.76 | 12.79 | 80.39 | 0.010 | -0.248 |
| *L. melli* | 11,200 | 38.47 | 9.16 | 38.16 | 14.18 | 76.63 | 0.003 | -0.201 |
| *M. pallidicosta* | 11,205 | 39.25 | 8.67 | 39.29 | 12.78 | 78.54 | 0.000 | -0.191 |
| **tRNA** |  |  |  |  |  |  |  |  |
| *Odontodes seranensis* | 1476 | 39.91 | 8.27 | 41.33 | 10.5 | 81.24 | -0.017 | -0.119 |
| *Hyblaea puera* | 1471 | 41.4 | 8.16 | 39.7 | 10.74 | 81.1 | 0.020 | -0.136 |
| *Actinotia polyodon* | 1472 | 41.03 | 8.08 | 41.03 | 9.85 | 82.06 | 0 | -0.098 |
| *Oraesia emarginata* | 1479 | 41.45 | 7.98 | 40.43 | 10.14 | 81.88 | 0.012 | -0.119 |
| *Eudocima salaminia* | 1459 | 40.64 | 9.25 | 40.99 | 9.12 | 81.63 | -0.004 | 0.007 |
| *E. phalonia* | 1462 | 40.90 | 8.27 | 40.49 | 10.32 | 81.39 | 0.005 | -0.110 |
| *P. flavescens* | 1485 | 41.62 | 7.81 | 40.61 | 9.97 | 82.22 | 0.012 | -0.121 |
| *O. lunifer* | 1666 | 41.78 | 7.33 | 39.86 | 11.04 | 81.63 | 0.023 | -0.202 |
| *H. cunea* | 1474 | 41.86 | 7.87 | 39.89 | 10.38 | 81.75 | 0.024 | -0.138 |
| 1. *formosae* | 1466 | 40.45 | 7.98 | 40.24 | 11.32 | 80.69 | 0.002 | -0.173 |
| *Doa* sp. | 1456 | 41.55 | 8.17 | 39.62 | 10.64 | 81.18 | 0.023 | -0.131 |
| *N. arctata albofasciata* | 1440 | 40.90 | 8.05 | 40.48 | 10.55 | 81.38 | 0.005 | -0.134 |
| *L. dispar* | 1469 | 41.66 | 7.96 | 39.35 | 11.03 | 81.01 | 0.029 | -0.162 |
| *G. menyuanensis* | 1504 | 41.29 | 7.38 | 41.76 | 9.57 | 83.05 | -0.006 | -0.129 |
| *H. armigera* | 1471 | 41.40 | 8.15 | 40.38 | 10.06 | 81.78 | 0.012 | -0.104 |
| *H. puntigera* | 1478 | 41.67 | 8.18 | 40.18 | 9.94 | 81.85 | 0.018 | -0.097 |
| *H. assulta* | 1471 | 41.33 | 8.29 | 40.31 | 10.06 | 81.64 | 0.012 | -0.096 |
| *A. ipsilon* | 1475 | 41.15 | 8.13 | 40.47 | 10.23 | 81.62 | 0.008 | -0.114 |
| *A. segetum* | 1471 | 40.72 | 8.22 | 40.85 | 10.19 | 81.57 | -0.001 | -0.107 |
| *S. litura* | 1473 | 42.23 | 7.94 | 39.58 | 10.25 | 81.81 | 0.032 | -0.127 |
| *S. exigua* | 1468 | 41.62 | 8.03 | 40.05 | 10.28 | 81.67 | 0.019 | -0.122 |
| *C. agnata* | 1472 | 41.10 | 8.22 | 40.28 | 10.39 | 81.38 | 0.010 | -0.116 |
| *C. limbirena* | 1466 | 40.38 | 8.18 | 41.33 | 10.09 | 81.71 | -0.011 | -0.104 |
| *E. adulatricoides* | 1475 | 41.35 | 8.06 | 40.47 | 10.10 | 81.82 | 0.010 | -0.111 |
| *S. inferens* | 1478 | 40.86 | 8.25 | 40.66 | 10.21 | 81.52 | 0.002 | -0.106 |
| *N. pronuba* | 1468 | 40.53 | 8.51 | 40.66 | 10.28 | 81.19 | 0.001 | -0.094 |
| *A. psi* | 1402 | 42.22 | 7.98 | 39.37 | 8.27 | 81.59 | 0.034 | -0.131 |
| *Striacosta albicosta* | 1486 | 40.58 | 8.48 | 40.44 | 10.50 | 81.02 | 0.002 | -0.106 |
| *M. separata* | 1473 | 41.07 | 8.15 | 40.73 | 10.05 | 81.81 | 0.004 | -0.104 |
| *R. prominens* | 1464 | 40.77 | 8.26 | 41.12 | 9.83 | 81.89 | -0.004 | -0.086 |
| *Catocala deuteronympha* | 1478 | 41.27 | 7.84 | 40.52 | 10.35 | 81.79 | 0.009 | -0.137 |
| *Asota plana* | 1457 | 40.28 | 8.23 | 40.70 | 10.77 | 80.98 | -0.005 | -0.133 |
| *P. curvicornata* | 1460 | 40.41 | 8.15 | 40.95 | 10.47 | 81.36 | -0.006 | -0.125 |
| *P. songi* | 1469 | 41.10 | 8.23 | 40.09 | 10.48 | 81.19 | 0.013 | -0.120 |
| *S. scintillans* | 1475 | 40.74 | 7.59 | 41.76 | 9.89 | 82.50 | -0.012 | -0.131 |
| *S. subcarnea* | 1458 | 40.53 | 8.71 | 39.71 | 11.04 | 80.24 | 0.010 | -0.118 |
| *S. frugiperda* | 1482 | 41.76 | 7.82 | 40.62 | 9.78 | 82.38 | 0.013 | -0.011 |
| *V. virilis* | 1456 | 41.00 | 8.10 | 40.52 | 10.37 | 81.52 | 0.005 | -0.122 |
| *A lepigone* | 1465 | 40.0 | 8.19 | 41.43 | 10.38 | 81.43 | -0.017 | -0.117 |
| *C dominula* | 1462 | 41.04 | 8.0 | 41.04 | 9.92 | 82.08 | 0 | -0.10 |
| *C. anachoreta* | 1469 | 40.7 | 8.11 | 40.57 | 10.62 | 81.27 | 0.001 | -0.13 |
| *C. anastomosis* | 1490 | 40.87 | 8.05 | 40.74 | 10.34 | 81.61 | 0.001 | -0.124 |
| *Gyana* sp. | 1476 | 40.79 | 7.93 | 40.92 | 10,37 | 81.71 | -0.001 | -0.133 |
| *E. cryptosticta* | 1467 | 41.58 | 8.11 | 39.88 | 10.43 | 81.46 | 0.020 | -0.125 |
| *E. pseudoconspersa* | 1466 | 41.41 | 8.19 | 40.18 | 10.23 | 81.59 | 0.015 | -0.11 |
| *E. similis* | 1480 | 41.69 | 7.7 | 40.54 | 10.07 | 82.23 | 0.013 | -0.133 |
| *G. argentata* | 1469 | 41.32 | 8.24 | 40.23 | 10.21 | 81.55 | 0.013 | -0.107 |
| *G. geometrica* | 1481 | 41.19 | 8.24 | 40.24 | 10.33 | 81.43 | 0.011 | -0.112 |
| *G. aureata* | 1511 | 41.23 | 7.48 | 41.96 | 9.33 | 83.19 | -0.008 | -0.110 |
| *G. juizhiensis* | 1493 | 41.33 | 7.43 | 41.53 | 9.71 | 82.86 | -0.002 | -0.132 |
| *H. gelotopoen* | 1474 | 41.59 | 8.14 | 40.09 | 10.18 | 81.68 | 0.018 | -0.111 |
| *G. minora* | 1509 | 41.15 | 7.36 | 42.15 | 9.34 | 83.3 | -0.011 | -0.119 |
| *G. qinghaiensis* | 1495 | 41.27 | 7.29 | 41.74 | 9.7 | 83.01 | -0.005 | -0.417 |
| *G. qumalensis* | 1498 | 41.19 | 7.34 | 41.52 | 9.95 | 82.71 | -0.004 | -0.150 |
| *G. ruorgensis* | 1510 | 41.39 | 7.35 | 41.85 | 9.4 | 83.24 | -0.005 | -0.122 |
| *H. lentalis* | 1540 | 40.16 | 8.05 | 41.42 | 10.37 | 81.58 | -0.015 | -0.126 |
| *L. alpherakii* | 1520 | 41.41 | 7.39 | 41.61 | 9.59 | 83.02 | -0.002 | -0.129 |
| *L. melli* | 1486 | 40.58 | 8.55 | 40.24 | 10.63 | 80.82 | 0.004 | -0.108 |
| *M. pallidicosta* | 1473 | 40.94 | 8.01 | 40.39 | 10.66 | 81.33 | 0.006 | -0.418 |
| **rRNA** |  |  |  |  |  |  |  |  |
| *Odontodes seranensis* | 2118 | 40.89 | 4.91 | 43.86 | 10.34 | 84.75 | -0.035 | -0.356 |
| *Hyblaea puera* | 2038 | 41.81 | 5.0 | 42.44 | 10.75 | 84.25 | -0.007 | -0.364 |
| *Actinotia polyodon* | 2148 | 40.88 | 4.93 | 43.95 | 10.24 | 84.95 | -0.036 | -0.483 |
| *Oraesia emarginata* | 2054 | 40.99 | 5.01 | 43.18 | 10.81 | 84.17 | -0.026 | -0.366 |
| *Eudocima salaminia* | 2036 | 41.26 | 4.91 | 42.90 | 10.05 | 84.24 | -0.020 | -0.376 |
| *E. phalonia* | 2212 | 41.50 | 4.74 | 43.49 | 10.26 | 84.99 | -0.023 | -0.367 |
| *P. flavescens* | 2198 | 41.31 | 4.73 | 44.04 | 9.92 | 85.35 | -0.032 | -0.354 |
| *O. lunifer* | 2157 | 41.96 | 4.82 | 40.19 | 13.03 | 82.15 | 0.022 | -0.460 |
| *H. cunea* | 2234 | 42.08 | 4.92 | 42.75 | 10.25 | 84.83 | -0.008 | -0.351 |
| 1. *formosae* | 2163 | 38.93 | 4.72 | 44.85 | 11.51 | 83.77 | -0.071 | -0.418 |
| *Doa* sp. | 2146 | 41.61 | 4.98 | 42.54 | 10.85 | 84.15 | -0.01 | -0.370 |
| *N. arctata albofasciata* | 2058 | 40.91 | 4.81 | 43.34 | 10.93 | 84.25 | -0.028 | -0.388 |
| *L. dispar* | 2150 | 42.79 | 4.79 | 41.81 | 10.60 | 84.60 | 0.012 | -0.377 |
| *G. menyuanensis* | 2311 | 4189 | 4.28 | 42.84 | 10.99 | 84.73 | -0.011 | -0.439 |
| *H. armigera* | 2155 | 41.62 | 4.91 | 43.34 | 10.11 | 84.96 | -0.020 | -0.345 |
| *H. puntigera* | 2161 | 41.87 | 4.81 | 43.35 | 9.94 | 85.22 | -0.017 | -0.347 |
| *H. assulta* | 2156 | 41.46 | 5.19 | 43.36 | 9.97 | 84.82 | -0.022 | -0.314 |
| *A. ipsilon* | 2133 | 41.49 | 5.06 | 43.45 | 9.98 | 84.94 | -0.023 | -0.327 |
| *A. segetum* | 2155 | 41.48 | 4.91 | 43.57 | 10.02 | 85.05 | -0.024 | -0.341 |
| *S. litura* | 2218 | 42.65 | 4.73 | 42.34 | 10.28 | 84.99 | 0.004 | -0.370 |
| *S. exigua* | 2165 | 42.35 | 4.66 | 42.63 | 10.34 | 84.98 | -0.003 | -0.378 |
| *C. agnata* | 2156 | 39.93 | 5.10 | 44.57 | 10.38 | 84.50 | -0.054 | -0.341 |
| *C. limbirena* | 2089 | 39.39 | 5.12 | 45.28 | 10.19 | 84.67 | -0.069 | -0.331 |
| *E. adulatricoides* | 2169 | 41.53 | 4.97 | 42.87 | 10.60 | 84.40 | -0.015 | -0.360 |
| *S. inferens* | 2146 | 41.51 | 4.75 | 42.45 | 11.27 | 83.96 | -0.011 | 0.406 |
| *N. pronuba* | 2144 | 40.95 | 4.89 | 44.12 | 10.02 | 85.07 | -0.037 | -0.347 |
| *A. psi* | 2143 | 41.62 | 4.99 | 41.90 | 11.47 | 83.52 | -0.003 | -0.393 |
| *Striacosta albicosta* | 2147 | 42.06 | 4.84 | 41.92 | 11.18 | 83.98 | 0.002 | -0.396 |
| *M. separata* | 2198 | 41.31 | 4.73 | 44.04 | 9.92 | 85.35 | -0032 | -0.354 |
| *R. prominens* | 2178 | 42.51 | 4.77 | 42.88 | 9.82 | 85.39 | -0.004 | -0.345 |
| *Catocala deuteronympha* | 2193 | 40.08 | 4.69 | 44.64 | 10.57 | 84.72 | -0.053 | -0.385 |
| *Asota plana* | 2172 | 41.43 | 4.83 | 43.18 | 10.54 | 84.61 | -0.020 | -0.371 |
| *P. curvicornata* | 2274 | 41.68 | 4.66 | 43.40 | 10.24 | 85.08 | -0.020 | -0.37 |
| *P. songi* | 2135 | 41.21 | 4.87 | 43.32 | 10.58 | 84.53 | -0.024 | -0.369 |
| *S. scinitillans* | 2080 | 41.58 | 4.85 | 43.99 | 9.56 | 85.57 | -0.028 | -0.326 |
| *S. subcarnea* | 2213 | 40.89 | 6.14 | 41.97 | 11.43 | 82.86 | -0.013 | -0.335 |
| *S. frugiperda* | 2125 | 41.92 | 4.84 | 42.40 | 10.82 | 84.32 | -0.005 | -0.381 |
| *V. virilis* | 2172 | 41.02 | 4.74 | 43.78 | 10.45 | 84.80 | -0.030 | -0.37 |
| *Athetis lepigone* | 2159 | 40.57 | 4.82 | 44.37 | 10.24 | 84.94 | 0.044 | -0.36 |
| *C. dominula* | 2154 | 41.23 | 4.87 | 43.31 | 10.58 | 84.54 | -0.024 | -0.36 |
| *C. anachoreta* | 2259 | 40.50 | 4.83 | 44.67 | 10.0 | 85.17 | -0.048 | -0.34 |
| *C. anastomosis* | 2186 | 41.26 | 4.76 | 43.78 | 10.20 | 85.04 | -0.02 | -0.36 |
| *Cyana* sp*.* | 2192 | 40.37 | 4.74 | 44.3 | 10.58 | 84.67 | -0.046 | -0.38 |
| *E. cryptosticta* | 2225 | 42.56 | 4.49 | 42.25 | 10.7 | 84.81 | 0.003 | -0.40 |
| *E. pseudoconspersa* | 2225 | 42.56 | 4.54 | 42.11 | 10.79 | 84.67 | 0.005 | -0.40 |
| *E. similis* | 2185 | 41.42 | 4.53 | 43.3 | 10.76 | 84.72 | -0.02 | -0.40 |
| *G. argentata* | 2165 | 40.60 | 4.76 | 45.13 | 9.52 | 85.73 | -0.05 | -0.33 |
| *G. geometrica* | 2147 | 42.29 | 4.94 | 42.2 | 10.57 | 84.49 | 0.001 | -0.36 |
| *G. aureata* | 2367 | 42.25 | 4.18 | 42.92 | 10.65 | 85.17 | -0.007 | -0.43 |
| *G. juizhiensis* | 2329 | 42.29 | 4.21 | 42.64 | 10.86 | 84.93 | -0.004 | -0.44 |
| *H. gelotopoeon* | 2167 | 41.49 | 4.80 | 43.29 | 10.43 | 84.78 | -0.021 | -0.36 |
| *G. minora* | 2384 | 42.32 | 4.19 | 42.95 | 10.53 | 85.27 | -0.021 | -0.43 |
| *G. qinghaiensis* | 2315 | 42.16 | 4.23 | 42.59 | 11.02 | 84.75 | -0.005 | -0.44 |
| *G. qumalensis* | 2388 | 42.00 | 4.23 | 43.59 | 10.18 | 85.59 | -0.018 | -0.41 |
| *G. ruorgensis* | 2390 | 42.34 | 4.10 | 42.97 | 10.59 | 85.31 | -0.007 | -0.44 |
| *H. lentalis* | 2207 | 40.55 | 4.58 | 44.04 | 10.83 | 84.59 | -0.041 | -0.40 |
| *L. alpherakii* | 2334 | 42.29 | 4.20 | 42.89 | 10.63 | 85.18 | -0.007 | -0.43 |
| *L. melli* | 2233 | 42.23 | 4.93 | 41.96 | 10.88 | 84.19 | 0.003 | -0.37 |
| *M. pallidicosta* | 2160 | 41.39 | 4.77 | 43.56 | 10.28 | 84.95 | 0.025 | -0.36 |
| **A+T-rich region** |  |  |  |  |  |  |  |  |
| *Odontodes seranensis* | 344 | 46.8 | 1.16 | 48.55 | 3.49 | 95.35 | -0.018 | -0.4 |
| *Hyblaea puera* | 437 | 47.14 | 1.14 | 50.11 | 1.6 | 97.25 | -0.030 | -0.166 |
| *Actinotia polyodon* | 341 | 43.99 | 2.05 | 50.73 | 3.23 | 94.72 | -0.071 | -0.222 |
| *Oraesia emarginata* | 287 | 46.69 | 2.09 | 46.69 | 4.53 | 93.38 | 0 | -0.368 |
| *Eudocima salaminia* | 436 | 47.94 | 5.04 | 44.95 | 2.06 | 92.89 | 0.032 | 0.420 |
| *E. phalonia* | 336 | 43.45 | 2.97 | 49.70 | 3.86 | 93.15 | -0.067 | -0.130 |
| *P. flavescens* | 541 | 42.14 | 2.22 | 49.72 | 5.91 | 91.86 | -0.083 | -0.454 |
| *O. lunifer* | 319 | 44.50 | 1.60 | 48.90 | 5.00 | 93.40 | -0.047 | -0.524 |
| *H. cunea* | 357 | 45.66 | 1.12 | 49.30 | 3.92 | 94.96 | -0.038 | -0.556 |
| 1. *formosae* | 484 | 42.97 | 2.89 | 49.79 | 4.33 | 92.76 | -0.073 | -0.2 |
| *Doa* sp. | 332 | 43.07 | 0.60 | 53.61 | 2.71 | 96.68 | -0.109 | -0.636 |
| *N. arctata albofasciata* | 401 | 43.39 | 1.99 | 50.37 | 4.23 | 93.76 | -0.074 | -0.36 |
| *L. dispar* | 435 | 45.29 | 1.61 | 50.80 | 2.30 | 96.09 | -0.057 | -0.176 |
| *G. menyuanensis* | 449 | 43.65 | 2.45 | 49.67 | 4.23 | 93.32 | -0.065 | -0.266 |
| *H. armigera* | 329 | 44.37 | 1.21 | 50.75 | 3.64 | 95.12 | -0.067 | -0.5 |
| *H. puntigera* | 328 | 45.12 | 1.21 | 51.21 | 2.43 | 96.33 | -0.063 | -0.333 |
| *H. assulta* | 329 | 44.37 | 1.21 | 50.45 | 3.95 | 94.82 | 0.064 | -0.529 |
| *A. ipsilon* | 344 | 46.22 | 1.45 | 48.83 | 3.48 | 95.05 | -0.027 | 0.411 |
| *A. segetum* | 345 | 46.08 | 0.86 | 48.40 | 4.63 | 94.48 | -0.024 | -0.684 |
| *S. litura* | 326 | 46.63 | 2.15 | 47.24 | 3.99 | 93.87 | -0.006 | -0.300 |
| *S. exigua* | 335 | 42.98 | 2.68 | 50.44 | 3.88 | 93.42 | -0.079 | -0.181 |
| *C. agnata* | 334 | 46.70 | 1.49 | 46.70 | 5.08 | 93.4 | 0 | -0.545 |
| *C. limbirena* | 422 | 46.91 | 2.36 | 46.20 | 4.50 | 93.11 | 0.007 | -0.473 |
| *E. adulatricoides* | 341 | 46.04 | 2.63 | 46.62 | 4.69 | 92.66 | -0.006 | -0.28 |
| *S. inferens* | 314 | 43.31 | 1.27 | 52.54 | 2.86 | 95.85 | -0.095 | -0.384 |
| *N. pronuba* | 330 | 44.24 | 2.12 | 49.09 | 4.54 | 93.33 | -0.051 | -0.363 |
| *A. psi* | 354 | 44.63 | 2.54 | 47.74 | 5.08 | 92.37 | -0.033 | -0.333 |
| *Striacosta albicosta* | 385 | 43.12 | 1.30 | 49.87 | 5.71 | 92.99 | -0.073 | -0.629 |
| *M. separata* | 372 | 44.62 | 2.42 | 49.73 | 3.23 | 94.35 | -0.054 | -0.143 |
| *R. prominens* | 343 | 44.02 | 2.33 | 49.59 | 1.11 | 93.58 | -0.059 | 0.333 |
| *Catocala deuteronympha* | 390 | 42.30 | 2.82 | 47.94 | 6.92 | 90.24 | -0.062 | -0.421 |
| *Asota plana* | 331 | 45.61 | 1.20 | 48.94 | 4.22 | 94.55 | -0.035 | -0.55 |
| *M. separata* | 372 | 44.62 | 2.41 | 49.73 | 3.24 | 94.35 | -0.05 | -0.14 |
| *O. lunifer* | 319 | 44.51 | 1.56 | 48.90 | 5.01 | 93.41 | -0.04 | -0.52 |
| *P. curvicornata* | 340 | 40.29 | 2.05 | 51.17 | 6.47 | 91.46 | -0.11 | -0.51 |
| *P. flavescens* | 541 | 42.14 | 2.21 | 49.72 | 5.91 | 91.86 | -0.08 | -0.45 |
| *P. songi* | 340 | 42.35 | 2.94 | 50.88 | 3.82 | 93.23 | -0.09 | -0.13 |
| *S. scintillans* | 445 | 42.47 | 0.89 | 50.33 | 6.29 | 92.80 | -0.08 | -0.75 |
| *S. subcarnea* | 355 | 43.94 | 1.12 | 49.57 | 5.35 | 93.51 | -0.06 | -0.65 |
| *S. frugiperda* | 330 | 45.75 | 1.51 | 47.57 | 5.15 | 93.32 | -0.01 | -0.54 |
| *S. litura* | 326 | 46.93 | 2.12 | 46.93 | 3.93 | 93.86 | 0 | -0.30 |
| *V. virilis* | 362 | 44.47 | 1.10 | 50.55 | 3.86 | 95.02 | -0.06 | -0.55 |
| *A. lepigone* | 542 | 44.46 | 1.11 | 49.63 | 4.8 | 94.09 | -0.05 | -0.625 |
| *C. dominula* | 486 | 39.92 | 10.7 | 35.19 | 14.2 | 75.11 | 0.063 | -0.80 |
| *C. anachoreta* | 347 | 42.94 | 2.88 | 48.99 | 5.19 | 91.93 | -0.065 | -0.28 |
| *C. anastomosis* | 325 | 41.54 | 1.85 | 51.69 | 4.92 | 93.23 | -0.108 | -0.454 |
| *E. cryptosticta* | 389 | 43.44 | 2.31 | 50.64 | 3.6 | 94.08 | -0.076 | -0.208 |
| *E. pseudoconspersa* | 388 | 43.56 | 2.32 | 50.26 | 3.87 | 93.82 | -0.07 | -0.25 |
| *E. similis* | 236 | 43.64 | 2.12 | 49.58 | 4.66 | 93.22 | -0.063 | -0.375 |
| *G. argentata* | 340 | 43.24 | 1.47 | 52.06 | 3.24 | 95.3 | -0.092 | -0.375 |
| *G. geometrica* | 407 | 46.19 | 2.7 | 45.95 | 5.16 | 92.14 | 0.002 | -0.312 |
| *G. aureata* | 446 | 43.50 | 2.69 | 49.1 | 4.71 | 92.6 | -0.060 | -0.27 |
| *G. juizhiensis* | 450 | 43.33 | 2.67 | 48.44 | 5.56 | 91.77 | -0.05 | -0.35 |
| *G. minora* | 449 | 43.20 | 2.67 | 49.44 | 4.67 | 92.64 | -0.06 | -0.272 |
| *G. qinghaiensis* | 449 | 42.76 | 3.11 | 48.55 | 5.56 | 91.31 | -0.06 | -0.282 |
| *G. qumalensis* | 373 | 46.11 | 2.14 | 46.64 | 5.09 | 92.75 | -0.01 | -0.407 |
| *G. ruorgensis* | 449 | 43.42 | 2.67 | 48.99 | 4.89 | 92.41 | -0.60 | -0.294 |
| *H. lentalis* | 331 | 44.41 | 1.20 | 50.75 | 3.62 | 95.16 | -0.06 | -0.500 |
| *L. alpherakii* | 449 | 44.76 | 2.22 | 55.00 | 4.67 | 99.76 | -0.03 | -0.034 |
| *L. melli* | 338 | 43.19 | 1.47 | 51.18 | 4.14 | 94.37 | -0.08 | -0.47 |
| *M. pallidicosta* | 328 | 45.12 | 1.82 | 48.47 | 4.57 | 93.59 | -0.03 | -0.42 |
