# Supplementary material for: Characterization of four mitochondrial genomes from superfamilies Noctuoidea and Hyblaeoidea with their phylogenetic implications
Source: Sci Rep. 2022 Nov 7;12:18926. doi: 10.1038/s41598-022-21502-y (PMC9640664; doi:10.1038/s41598-022-21502-y)
Supplement: Supplementary file 6 — Supplementary Information 6. [file 41598_2022_21502_MOESM6_ESM.docx]

**Supplementary Table 2**

***Actinotia polyodon***

| Codon | N | RSCU | Codon | N | RSCU | Codon | N | RSCU | Codon | N | RSCU |
| --- | --- | --- | --- | --- | --- | --- | --- | --- | --- | --- | --- |
| UUU(F) | 292 | 1.61 | UCU(S) | 52 | 1.57 | UAU(Y) | 189 | 1.72 | UGU(C) | 27 | 1.54 |
| UUC(F) | 70 | 0.39 | UCC(S) | 20 | 0.60 | UAC(Y) | 31 | 0.28 | UGC(C) | 8 | 0.46 |
| UUA(L) | 269 | 3.44 | UCA(S) | 85 | 2.56 | UAA (*) | 197 | 1.94 | UGA (*) | 57 | 0.56 |
| UUG(L) | 32 | 0.41 | UCG(S) | 9 | 0.27 | UAG (*) | 51 | 0.50 | UGG(W) | 10 | 1.00 |
| CUU(L) | 68 | 0.87 | CCU (P | 48 | 1.85 | CAU(H) | 71 | 1.78 | CGU(R) | 7 | 0.49 |
| CUC(L) | 12 | 0.15 | CCC(P) | 13 | 0.50 | CAC(H) | 9 | 0.23) | CGC(R) | 3 | 0.21 |
| CUA(L) | 74 | 0.95 | CCA(P) | 41 | 1.58 | CAA(Q) | 67 | 1.49 | CGA(R) | 22 | 1.55 |
| CUG(L) | 14 | 0.18 | CCG(P) | 2 | 0.08 | CAG(Q) | 23 | 0.51 | CGG(R) | 1 | 0.07 |
| AUU(I) | 325 | 1.60 | ACU(T) | 56 | 1.62 | AAU(N) | 288 | 1.70 | AGU(S) | 20 | 0.60 |
| AUC(I) | 52 | 0.26 | ACC(T) | 18 | 0.52 | AAC(N) | 50 | 0.30 | AGC(S) | 13 | 0.39 |
| AUA(I) | 232 | 1.14 | ACA(T) | 60 | 1.74 | AAA(K) | 251 | 1.76 | AGA(R) | 34 | 2.40 |
| AUG(M) | 32 | 1.00 | ACG(T) | 4 | 0.12 | AAG (K | 34 | 0.24 | AGG(R) | 18 | 1.27 |
| GUU(V) | 22 | 1.54 | GCU(A) | 40 | 2.25 | GAU(D) | 57 | 1.68 | GGU(G) | 19 | 0.87 |
| GUC(V) | 1 | 0.07 | GCC(A) | 2 | 0.11 | GAC(D) | 11 | 0.32 | GGC(G) | 0 | 0.00 |
| GUA(V) | 32 | 2.25 | GCA(A) | 29 | 1.63 | GAA(E) | 77 | 1.66 | GGA(G) | 64 | 2.94 |
| GUG(V) | 2 | 0.14 | GCG(A) | 0 | 0.00 | GAG(E) | 16 | 0.34 | GGG(G) | 4 | 0.18 |

Average# codons= 3737

***Hyblaea puera***

| Codon | N | RSCU | Codon | N | RSCU | Codon | N | RSCU | Codon | N | RSCU |
| --- | --- | --- | --- | --- | --- | --- | --- | --- | --- | --- | --- |
| UUU(F) | 280 | 1.76 | UCU(S) | 63 | 2.04 | UAU(Y) | 120 | 1.64 | UGU(C) | 17 | 1.79 |
| UUC(F) | 39 | 0.24 | UCC(S) | 4 | 0.13 | UAC(Y) | 26 | 0.36 | UGC(C) | 2 | 0.21 |
| UUA(L) | 290 | 3.54 | UCA(S) | 96 | 3.11 | UAA(*) | 141 | 1.78 | UGA(*) | 68 | 0.86 |
| UUG(L) | 25 | 0.31 | UCG(S) | 3 | 0.10 | UAG(*) | 28 | 0.35 | UGG(W) | 2 | 1.00 |
| CUU(L) | 80 | 0.98 | CCU(P) | 76 | 2.17 | CAU(H) | 68 | 1.66 | CGU(R) | 5 | 0.45 |
| CUC(L) | 10 | 0.12 | CCC(P) | 17 | 0.49 | CAC(H) | 14 | 0.34 | CGC(R) | 0 | 0.00 |
| CUA(L) | 75 | 0.92 | CCA(P) | 47 | 1.34 | CAA(Q) | 81 | 1.72 | CGA(R) | 33 | 2.96 |
| CUG(L) | 11 | 0.13 | CCG(P) | 0 | 0.00 | CAG(Q) | 13 | 0.28 | CGG(R) | 1 | 0.09 |
| AUU(I) | 370 | 1.66 | ACU(T) | 86 | 2.08 | AAU(N) | 274 | 1.86 | AGU(S) | 17 | 0.55 |
| AUC(I) | 37 | 0.17 | ACC(T) | 7 | 0.17 | AAC(N) | 20 | 0.14 | AGC(S) | 2 | 0.06 |
| AUA(I) | 261 | 1.17 | ACA(T) | 69 | 1.67 | AAA(K) | 272 | 1.94 | AGA(R) | 27 | 2.42 |
| AUG(M) | 24 | 1.00 | ACG(T) | 3 | 0.07 | AAG(K) | 9 | 0.06 | AGG(R) | 1 | 0.09 |
| GUU(V) | 44 | 1.71 | GCU(A) | 54 | 2.14 | GAU(D) | 73 | 1.66 | GGU(G) | 23 | 0.78 |
| GUC(V) | 10 | 0.39 | GCC(A) | 3 | 0.12 | GAC(D) | 15 | 0.34 | GGC(G) | 3 | 0.10 |
| GUA(V) | 44 | 1.71 | GCA(A) | 44 | 1.74 | GAA(E) | 90 | 1.68 | GGA(G) | 88 | 2.98 |
| GUG(V) | 5 | 0.19 | GCG(A) | 0 | 0.00 | GAG(E) | 17 | 0.32 | GGG(G) | 4 | 0.14 |

Average# codons= 3731

***Odontodes seranensis***

| Codon | N | RSCU | Codon | N | RSCU | Codon | N | RSCU | Codon | N | RSCU |
| --- | --- | --- | --- | --- | --- | --- | --- | --- | --- | --- | --- |
| UUU(F) | 276 | 1.64 | UCU(S) | 104 | 2.34 | UAU(Y) | 200 | 1.67 | UGU(C) | 17 | 1.42 |
| UUC(F) | 60 | 0.36 | UCC(S) | 34 | 0.76 | UAC(Y) | 40 | 0.33 | UGC(C) | 7 | 0.58 |
| UUA(L) | 257 | 3.53 | UCA(S) | 76 | 1.71 | UAA(*) | 228 | 2.16 | UGA(*) | 65 | 0.62 |
| UUG(L) | 41 | 0.56 | UCG(S) | 12 | 0.27 | UAG(*) | 23 | 0.22 | UGG(W) | 10 | 1.00 |
| CUU(L) | 62 | 0.85 | CCU(P) | 48 | 1.88 | CAU(H | 52 | 1.60 | CGU(R) | 6 | 0.35 |
| CUC(L) | 20 | 0.27 | CCC(P) | 19 | 0.75 | CAC(H) | 13 | 0.40 | CGC(R) | 0 | 0.00 |
| CUA(L) | 51 | 0.70 | CCA(P) | 34 | 1.33 | CAA(Q) | 63 | 1.59 | CGA(R) | 19 | 1.11 |
| CUG(L) | 6 | 0.08 | CCG(P | 1 | 0.04 | CAG(Q) | 16 | 0.41 | CGG(R) | 2 | 0.12 |
| AUU(I) | 332 | 1.62 | ACU(T | 54 | 1.32 | AAU(N) | 270 | 1.76 | AGU(S) | 17 | 0.38 |
| AUC(I) | 36 | 0.18 | ACC(T) | 48 | 1.17 | AAC(N) | 36 | 0.24 | AGC(S) | 24 | 0.54 |
| AUA(I) | 246 | 1.20 | ACA(T) | 52 | 1.27 | AAA(K) | 235 | 1.89 | AGA(R) | 58 | 3.38 |
| AUG(M) | 26 | 1.00 | ACG(T | 10 | 0.24 | AAG(K) | 14 | 0.11 | AGG(R) | 18 | 1.05 |
| GUU(V) | 28 | 1.40 | GCU(A) | 42 | 2.18 | GAU(D) | 58 | 1.61 | GGU(G) | 27 | 1.08 |
| GUC(V) | 5 | 0.25 | GCC(A) | 7 | 0.36 | GAC(D) | 14 | 0.39 | GGC(G) | 2 | 0.08 |
| GUA(V) | 44 | 2.20 | GCA(A) | 28 | 1.45 | GAA(E) | 56 | 1.62 | GGA(G) | 67 | 2.68 |
| GUG(V) | 3 | 0.15 | GCG(A | 0 | 0.00 | GAG(E) | 13 | 0.38 | GGG(G) | 4 | 0.16 |

Average# codons= 3736

***Oraesia emarginata***

| Codon | N | RSCU | Codon | N | RSCU | Codon | N | RSCU | Codon | N | RSCU |
| --- | --- | --- | --- | --- | --- | --- | --- | --- | --- | --- | --- |
| UUU(F) | 274 | 1.63 | UCU(S) | 58 | 1.44 | UAU(Y) | 194 | 1.67 | UGU(C) | 38 | 1.43 |
| UUC(F) | 62 | 0.37 | UCC(S) | 29 | 0.72 | UAC(Y) | 38 | 0.33 | UGC(C) | 15 | 0.57 |
| UUA(L) | 273 | 3.38 | UCA(S | 91 | 2.27 | UAA(*) | 172 | 1.91 | UGA(*) | 53 | 0.59 |
| UUG(L) | 35 | 0.43 | UCG(S) | 10 | 0.25 | UAG(*) | 45 | 0.50 | UGG(W) | 19 | 1.00 |
| CUU(L) | 61 | 0.75 | CCU(P) | 43 | 1.50 | CAU(H) | 60 | 1.60 | CGU(R) | 10 | 0.64 |
| CUC(L) | 27 | 0.33 | CCC(P) | 24 | 0.83 | CAC(H) | 15 | 0.40 | CGC(R) | 0 | 0.00 |
| CUA(L) | 75 | 0.93 | CCA(P) | 46 | 1.60 | CAA(Q) | 63 | 1.52 | CGA(R) | 15 | 0.96 |
| CUG(L) | 14 | 0.17 | CCG(P) | 2 | 0.07 | CAG(Q) | 20 | 0.48 | CGG(R) | 2 | 0.13 |
| AUU(I) | 294 | 1.52 | ACU(T) | 50 | 1.43 | AAU(N) | 268 | 1.46 | AGU(S) | 29 | 0.72 |
| AUC(I) | 70 | 0.36 | ACC(T) | 26 | 0.74 | AAC(N) | 98 | 0.54 | AGC(S) | 24 | 0.60 |
| AUA(I) | 215 | 1.11 | ACA(T | 58 | 1.66 | AAA(K) | 246 | 1.70 | AGA(R) | 34 | 2.17 |
| AUG(M) | 32 | 1.00 | ACG(T) | 6 | 0.17 | AAG(K) | 44 | 0.30 | AGG(R) | 33 | 2.11 |
| GUU(V) | 25 | 1.72 | GCU(A) | 35 | 2.50 | GAU(D) | 42 | 1.68 | GGU(G) | 25 | 1.22 |
| GUC(V) | 1 | 0.07 | GCC(A) | 6 | 0.43 | GAC(D) | 8 | 0.32 | GGC(G) | 1 | 0.05 |
| GUA(V) | 29 | 2.00 | GCA(A) | 15 | 1.07 | GAA(E) | 62 | 1.75 | GGA(G) | 55 | 2.68 |
| GUG(V) | 3 | 0.21 | GCG(A) | 0 | 0.00 | GAG(E) | 9 | 0.25 | GGG(G | 1 | 0.05 |

Average# codons= 3727
